# A compendium of genome-wide sequence reads from NBS (nucleotide binding site) domains of resistance genes in the common potato

**DOI:** 10.1038/s41598-020-67848-z

**Published:** 2020-07-09

**Authors:** Celine Prakash, Friederike Ch Trognitz, Peter Venhuizen, Arndt von Haeseler, Bodo Trognitz

**Affiliations:** 1 Center for Integrative Bioinformatics Vienna, Max F. Perutz Laboratories, University of Vienna and Medical University of Vienna, 1030 Vienna, Austria; 2 AIT Austrian Institute of Technology, Konrad-Lorenz-Straße 24, 3430 Tulln, Austria; 3 Department of Applied Genetics and Cell Biology, University of Natural Resources and Life Sciences - BOKU, Muthgasse 18, 1190 Vienna, Austria; 4 Max Planck Institute for Evolutionary Biology, Max Planck Research Group “Biological Clocks”, August-Thienemann-Strasse 2, 24306 Plön, Germany; 5 Bioinformatics and Computational Biology, Faculty of Computer Science, University of Vienna, 1090 Vienna, Austria; 6 Excellence in Breeding platform EiB, International Maize and Wheat Improvement Center CIMMYT, Carretera México-Veracruz, km. 45, El Batán, 56237 Texcoco, Mexico

**Keywords:** Data mining, Data publication and archiving, Databases, Genome informatics, Sequence annotation

## Abstract

SolariX is a compendium of DNA sequence tags from the nucleotide binding site (NBS) domain of disease resistance genes of the common potato, *Solanum tuberosum* Group Tuberosum. The sequences, which we call NBS tags, for nearly all NBS domains from 91 genomes—representing a wide range of historical and contemporary potato cultivars, 24 breeding programs and 200 years—were generated using just 16 amplification primers and high-throughput sequencing. The NBS tags were mapped to 587 NBS domains on the draft potato genome DM, where we detected an average, over all the samples, of 26 nucleotide polymorphisms on each locus. The total number of NBS domains observed, differed between potato cultivars. However, both modern and old cultivars possessed comparable levels of variability, and neither the individual breeder or country nor the generation or time appeared to correlate with the NBS domain frequencies. Our attempts to detect haplotypes (i.e., sets of linked nucleotide polymorphisms) frequently yielded more than the possible 4 alleles per domain indicating potential locus intermixing during the mapping of NBS tags to the DM reference genome. Mapping inaccuracies were likely a consequence of the differences of each cultivar to the reference genome used, coupled with high levels of NBS domain sequence similarity. We illustrate that the SolariX database is useful to search for polymorphism linked with NBS-LRR R gene alleles conferring specific disease resistance and to develop molecular markers for selection.

## Introduction

The common potato (*Solanum tuberosum* Group Tuberosum) was created in Europe. It has gone through considerable genetic bottlenecks, including the initial selection for adaptation to long-day prior to its first cropping in continental Europe^1^ as well as the epiphytotics of late blight in 1845–47 and wart around 1910 (reviewed in Ross^2^). Remains of first domesticated potatoes from Lima, Peru, were dated to 7,000 B.C.^3^. This Andean gene pool holds diploid, triploid, tetraploid and pentaploid varieties^4^. When compared with its Andean relatives, the European potato has a much narrower genetic base. The European potato owes its outstanding role as a worldwide popular staple food to two characteristics—its ability to produce high yield independent of the length of the day in many regions and its (ever expanding through breeding) resistance to late blight, cyst forming nematodes, wart (*Synchytrium endobioticum*), and several devastating viral diseases.

The defensive response of plants to pathogens is mediated through the protein products of resistance genes (R genes). To prevent yield losses resulting from disease susceptibility, it is essential that cultivars used in potato crop production contain disease resistance alleles. The European potato’s disease resistance is the result of at least 110 years of (pre-) breeding work to introgress R genes from wild and native cultivated genetic resources. One systematic approach at introgressing new resistances was made by R.N. Salaman, who added resistance to late blight^5^. This marked the start of a series of introgressions^6^ and the development of new, resistant cultivars until present^7, 8^. A common breeding scheme has been to cross a Group Tuberosum parental cultivar with a genebank accession of tuberous Solanum, either wild or domesticated, selected for a specific disease resistance. This is then followed by several rounds of backcrossing and selecting for individual, resistant progenies that also show other desirable characteristics of the Group Tuberosum parent. In potato, the total number of successful introgressions via crossing is not known, there may be from dozens up to over one hundred. Once stably introgressed, an R gene will be maintained in the breeding pool, and the number of different resistance alleles accumulated would increase with every new generation of potato cultivars. Potato breeding and selection has lagged behind other crops due to the complexity of this tetraploid, heterozygous plant. As such, characterizing the repertoire of R genes to facilitate development of disease resistant varieties is of great significance. *R1* was the first R gene characterized for resistance to late blight^9^ resulting from Salaman’s introgression work dating back to 1909^5^. *R1* resides on an insertional, introgressed locus of the Group Tuberosum potato^10^. The *R1* resistance allele has been found to be present in the genomes of multiple Group Tuberosum clones, which include some contemporary commercial potato varieties^11^.

As most R genes contain both nucleotide-binding site (NBS) and leucine-rich repeat (LRR) domains, conserved motifs from both NBS and LRR domains have been efficiently employed to identify the majority of plant NBS-LRR R genes^12–16^. Jupe et al.^13^, using close to 50,000, 120-mer biotinylated RNA oligos, enriched fragments putatively containing NBS-LRR domains from the genomic DNA of one Group Tuberosum cultivar and detected a total of 755 R genes containing NBS and LRR domains. They allocated 704 of these to the 12 established potato chromosomes and 51 to unanchored superscaffolds.

Jupe and colleagues’^13^ 755 R genes refer to the potato’s current, publicly available sequenced genome (version 4.03) of the diploid (doubled monoploid) *S. tuberosum* Group Phureja clone (clonal cultivar) DM 1–3 516 R44^15^. The homozygosity of this clone provided ease in sequencing of its genome. Group Phureja has been claimed to be a close relative of the tetraploid potato not only by taxonomy^17^ but also by genomic fine structure (Andean; Group Andigena and European; Group Tuberosum^13, 15^). Nonetheless, despite their close taxonomic relationship, the Group Phureja genome still differs somewhat from the Group Tuberosum genome. The individual used for sequencing, clone DM 1–3 516, may represent a rather small section of the whole genetic variability within the ancient cultivated potatoes comprising Group Phureja. *S. phureja* was, in fact, considered in pre-molecular taxonomy treatments as a separate species^4^.

Gene duplication has been considered to play an important role in the expansion of the R gene family. R genes are largely organized in clusters, also known as multiple-copy R loci that contain variable numbers of gene copies. Copy number variations differ on the cluster as well as the species level^12, 18^. The majority of NBS-LRR R genes in the DM genome occur stacked in clusters, where complete (putatively) functional genes alternate with incomplete ones. This is seen as a source of variation and a plant’s reservoir for producing new functional R alleles via frameshift recombination and DNA repair processes^19, 20^. Consequently, not only the number of R alleles, but the number of R genes may vary, even at cultivar level within a species^21^.

Recent high-throughput DNA sequencing methods open workable ways to elucidate the genetic diversity within the genepool of a single species. We employed Illumina HiSeq-2000 technology to mass-sequence highly conserved, characteristic 200 to 480- base pair (bp) fragments of the NBS domains of R genes, which we denominate NBS tags. These NBS tags were enriched from genomic fragments in a method similar to the NBS profiling of van der Linden et al.^16^—i.e. PCR amplification using a small number of priming oligonucleotides that target extremely conserved motifs of the NBS domain of R genes. These conserved motifs together with nearby highly polymorphic sequences, were used to efficiently survey the R gene pool for 91 genomes comprising both historical and contemporary potato cultivars. We detected in total 587 distinct NBS domains on the DM genome, 576 of these corresponded to NBS-LRR loci as laid out by Jupe et al.^13^ and 11 are additional. We provide the collection of the identified NBS domains of R genes on our SolariX website. This expands the current knowledge of the variability of potato R genes to a cultivar level resolution.

Our experimental questions were:

1. Is it possible to detect and (with Illumina HiSeq-2000 technology) distinguish individual R genes carrying NBS and LRR domains by inspecting NBS tags enriched via a small set of PCR primers that target conserved sections of the NBS domain?
  If this question can be answered positively, then:
2. How many NBS-LRR R genes can be detected, by this method, within the genepool of *S. tuberosum* Group Tuberosum?
3. Can individual alleles of R genes be distinguished through variations in sequences within their corresponding NBS tags and can we determine how many alleles there are?
4. Can specific patterns be discerned from how these R alleles are distributed across the groups of germplasm that are represented in the experimental materials (clones of different ages, generations and breeders)?
5. Can the NBS tagging method be used to efficiently discover R alleles responsible for specific resistances and can it be used to create selection markers for these alleles?

## Results

### Capture of NBS domains of the R gene pool using a limited number of PCR primers

#### Design of PCR primers for the P-loop, Kinase-2 and GLPL motifs of the NBS domain

**Figure 1:**
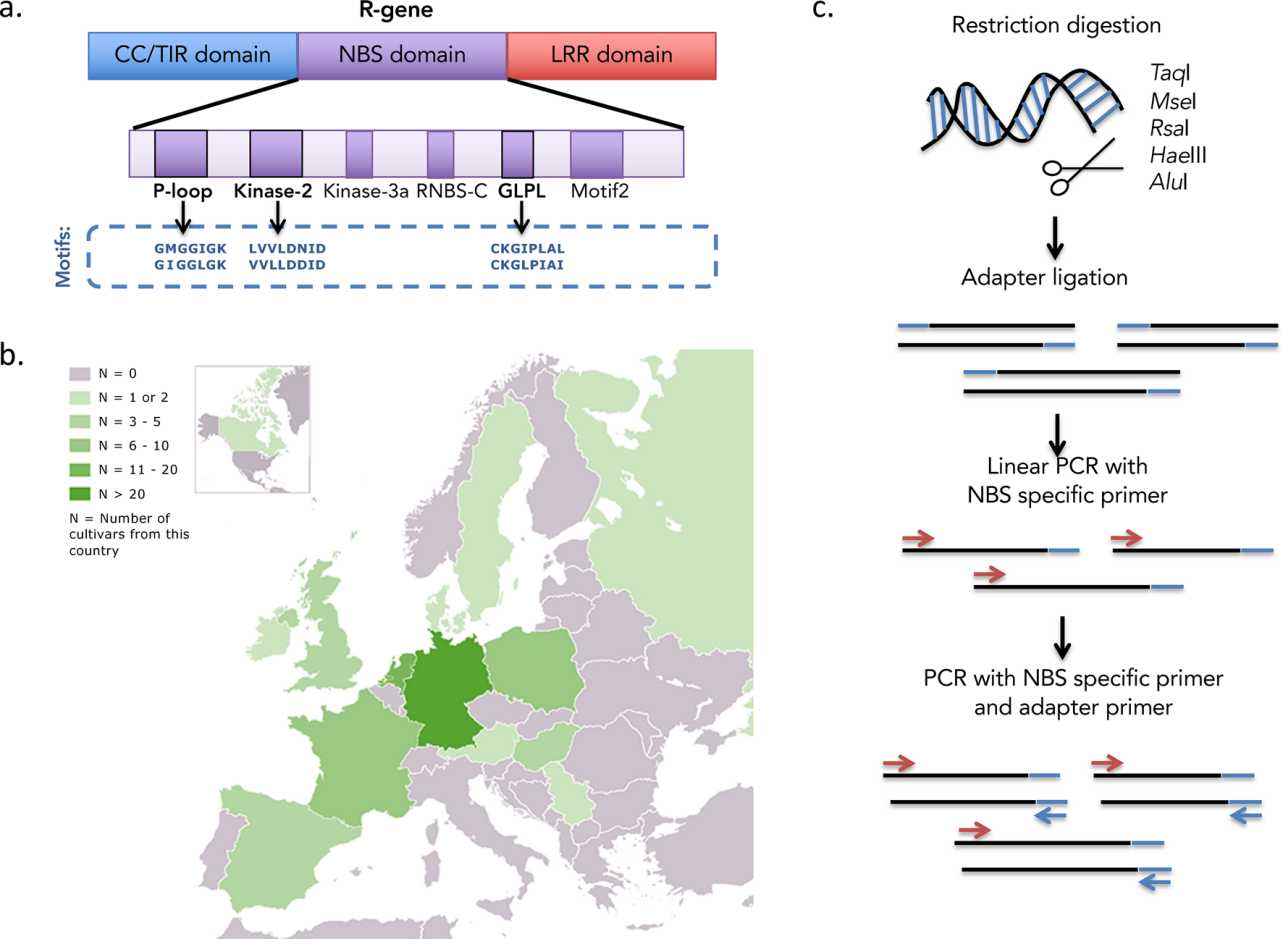
Capture of NBS domains of the R gene pool. (**a**) R-genes consist of a nucleotide binding (NB) and a leucine rich repeat (LRR) domain. They further have either the toll/interleukin-1 receptor (TIR) or the coiled-coil (CC) domain. Amplification primers were chosen to be complementary to one of the highly conserved P-loop, Kinase-2 and GLPL motifs within the NBS domain. PCR primers were designed using degeneracy at individual nucleotides to match the sequence diversity across 435 distinct NBS nucleotide sequences, obtained from tblastn searches in R genes mapped by Jupe et al.^13^. (**b**) The number of cultivars sequenced per country across Europe.[We adapted the map based on the File “Blank map of Europe cropped.svg” at https://commons.wikimedia.org/wiki/File:Blank_map_of_Europe_cropped.svg#file provided by ‘Maix’ (me@de) under a CC BY SA 3.0 Unported license (https://creativecommons.org/licenses/by-sa/3.0/legalcode).] The graphic in (**b**) is available under this same Creative Commons BY SA 3.0 Unported license. (**c**) The sequence of steps involved in targeted amplification of NBS domain containing genomic fragments.

The goal was to define a minimal set of primers that would amplify, out of genomic DNA, the maximum number of individual R gene alleles. The primers were chosen to be complementary to one of the highly conserved P-loop, Kinase-2 and GLPL motifs within the NBS domain of R genes (Fig. 1a). To design degenerate primers that could cover, as broadly as possible, the sequence diversity of the NBS domains of R genes present in the potato genome, tblastn searches across all 438 NBS-LRR R genes (the most current annotation at that point) mapped by Jupe et al.^12^ on the 12 established DM v 3.4 chromosomes for sequences of the NBS domain was performed. This returned 435 distinct nucleotide sequences, which were aligned, grouped by the similarity of their sequences and visually compared to pinpoint polymorphic positions within each of the three NBS-motifs. The highly conserved GLPL motif neighbors the highly polymorphic LRR domain of an R gene. Our primers targeting GLPL were chosen to allow for extension of the newly formed nucleotide chain from the NBS into the LRR domain (minimum 60 nucleotides) in order to include parts of the variable sequence, consequently increasing the individuality of the amplified fragment and the chance of unambiguous read mapping. Originally, we designed 24 PCR primers using degeneracy at individual nucleotides to match the sequence diversity across all 435 NBS-LRR loci on DM v 3.4. The functionality of these 24 primers was then verified by PCR on genomic DNA as a template. Fifteen of these 24 primers, as well as the NBS5 primer of van der Linden et al.^16^ that yielded abundant amplicons, were finally selected for the experiment (Table 1). As will be demonstrated further down, these in total 16 primers covered virtually all (complete and incomplete) R genes of the DM genome that carry at least one of the three NBS domain-specific motifs.

**Table 1:** Degenerate primers for selective PCR amplification of sequence tags from NB-LRR (Nucleotide Binding site and Leucine Rich Repeat) domains of R (Resistance) genes in the potato genome

Primer name		Primer Sequence 5′–3′	Annealing temperature (°C)	
kinase2_1		TNVTNITNITDGAYGAYNTNTGG	60	
kinase2-3		TNITTGTNCTTGATGAYRTNRA	55	
kinase2-4		TYSTYHTWGATGAYRTNTGG	55	
NBS5		YYTKRTHGTMITKGATGATGTITGG	55	
ploop_R1		GBATGGGNGGRCAAGGNAAAAC	60	
ploop_R2-2		GGHATDGSIGGHITNGGNAARAC	55	
ploop1		GGIGGINTRGGIAARACRAC	50	
ploop_PGT		CCIGGIACIGGIAARACIAC	60	
ploop_Sw5-2		TGGGYGGWNTIGGNAATACHAC	55	
ploop_Mi		GGNATGCCNGGNINIGGNAARAC	60	
GLP-2		GCDMMMGGNCWTCCDTTAGC	55	
GLPL2-2		TGYMARGGRYTKCCNYTDRYVVT	55	
GLPL2-3		TGYARRGGRYTDCCTYTDDYDVT	55	
GLPL3-1		TGYVRDGGNITRCCNYTDDC	55	
GLPL3-2		TGTGGMGGRTTGCCTCTCTTC	55	
Additional degenerate primers for amplification using a MiSeq apparatus (Illumina), of *R2*-like genes from the differential clone R2 and the samples representing the MT population				
R2ch4-F1	I*GTGTTTGAGATCAACTCTATTGCTAATG			55
R2ch4-F2	I*CAATTGTTGTATTGAGCGGACT			55
R2ch4-F3	I*GGAAAGATGTTGACCCTGTTG			55
R2ch4-F4	I*TGTGCAGTGATAACAGCTTCA			55
R2ch4-R2	I*GCTGCTAATGTTGTTTAGGGAGT			55
R2ch4-R3	I*TGGATCGAAGAACATAATTGACC			55
R2ch4-R4	I*AATGACTCTGCTTCCATTCTTG			55
R2ploop1	I*GAAWGGGBGGHTTRGGCAAGAC			55
R2ploop2	I*GCATGGGHGGWTTRGGCAAGAC			55
R2ploop3	I*GTATGGGMGGWTTRGGCAAGA			55
R2ploop4	I*GGATGGGTGGATTGGGCAAGA			55
R2GLPL	I*AGRAGAAAGTTGGKAYCTCTTT			55
R2Kinase2	I*GATGATBTRYGGMAKADWGAAG			55

Primer nucleotide sequence and individual annealing temperature for optimal amplification results.

I*- the Illumina adapter for MiSeq sequencing: TCGTCGGCAGCGTCAGATGTGTATAAGAGACAG.

The universal adapter primer for Illumina MiSeq was: GTCTCGTGGGCTCGGAGATGTGTATAAGAGACAGACTCGATTCTCAACCCGAAAG.

#### Potato cultivars and breeding clones used in SolariX

Ninety-six genomic DNA libraries were obtained from registered, modern as well as historical cultivars, advanced concurrent breeding clones, experimental cross populations segregating for disease resistance, and the wild non-tuberous *Solanum caripense* (CRP, for details see “Materials and methods”). The samples were obtained from a variety of European countries (Fig. 1b). Care was taken to include samples of the majority of original European potato breeding stocks, as is indicated in Table 2. Live samples of potato cultivars and breeding clones were kindly provided by European breeders and a genebank.

**Table 2:** Summary information on potato cultivars and breeding clones used in SolariX, and their breeders.

Country	No breeders	No cultivars
Austria	1	3
Canada	1	1
Denmark	1	2
France	4	12
Germany	5	27
Hungary	2	6
India	1	1
Ireland	1	1
Netherlands	4	11
Poland	2	7
Spain	2	9
UK	2	7
USA	2	2
Total	28	89

*Sources* European Cultivated Potato Database, SASA Edinburgh, UK, www.europotato.org/. For a detailed presentation of the materials, see Supplementary Table S2.

Five libraries, out of the 96, represent bulked DNA of disease resistant or susceptible segregants from our mapping populations. These were the progeny libraries in the following: For the tetraploid potato population MT^22^ ( M; cv. MF-II, T; cv. TPS67) segregating for R gene resistance to late blight, three libraries of progenies pooled by their distinct phenotype of resistance were included. As an outgroup, four libraries of diploid CRP were included, parental genotypes C1 (late blight resistant) and K4 (susceptible) and progenies Crp_pool_R and Crp_pool_S comprising either 10 late blight resistant (by the phenotype) or 10 susceptible individuals of a segregating CK progeny^23^.

#### Targeted amplification and sequencing of genome-wide fragments containing NBS domain motifs

The 16 NBS-motif primers were then used for targeted amplification in all 96 libraries after digestion with a 4-cutter restrictase and ligation of adapters as indicated in van der Linden et al.^16^ (see “Materials and methods” and Fig. 1c). In an initial linear PCR the NBS-motif primers were applied exclusively, allowing for enrichment of fragments containing NBS domain motifs. For subsequent exponential PCR the same NBS-motif primers were applied together with restriction site-specific adapter primers covering all restriction enzymes. PCR with all 16 primers was successful on all 96 DNA libraries, and per library, a single pooled sample containing the amplicons from all 16 primer-specific products was prepared. The random-sheared DNA fragments of each library were indexed (“Materials and methods”) and subjected to Illumina HiSeq-2000 (100-bp) paired-end sequencing.

### NBS domain annotation on the DM genome

Prior to any analysis of the sequencing data, an in silico annotation of the NBS domain within R genes was required to demarcate the regions that we had targeted for amplification and sequencing. Of the 704 NBS-LRR genes in the updated list by Jupe et al.^13^ on the 12 DM v 4.03 chromosomes, we annotated 576 regions with an NBS domain through a hidden Markov model (HMM) search similar to that done by Lozano et al.^14^ (see “Materials and methods”).

### Sequencing statistics of NBS-motif enriched libraries

#### Sequence read quality

Read sequence quality scores of mean 32.6 (median 35) for the first read in each read pair and mean 29.8 (median 35) for the second read in each pair were obtained. Although restriction enzyme digested genome libraries are known to have low complexity at the 5′ end of the sequenced fragment, we only observed a slightly lower per-base sequence quality in the first five bases sequenced, with the mean quality score ranging from 29.7 to 33.8 (median: 31 to 35, lower quartile: 28 to 33).

#### Sequence read mapping rates

**Figure 2:**
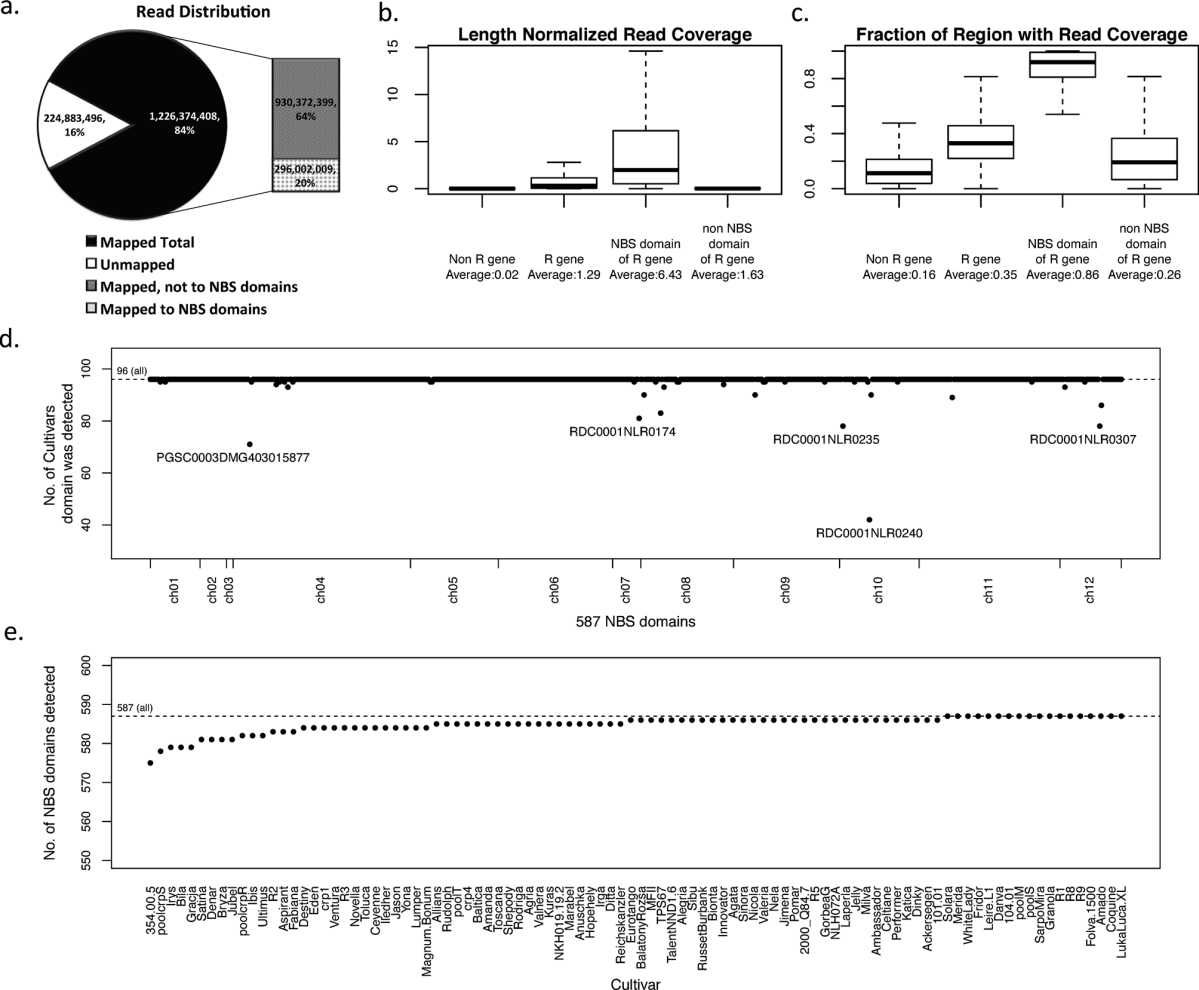
NBS domain sequencing statistics. (**a**) Percentage of total sequenced reads that were mapped to the doubled-monoploid (DM) potato clone version 4.03 genome and the distribution of the mapped reads within NBS domains. (**b**, **c**) Enriched sequencing coverage at NBS domains of R genes illustrates the specificity of the NBS-capture protocol—distributions of read coverage, normalized by length of the region (**b**) and fraction of bases in the region with non-zero read coverage (**c**) for all 96 sequencing libraries at annotated regions: Non-resistance genes, resistance genes, NB domains of R genes, Non NB domains of R genes (boxplots left to right). (**d**) NBS domain detection rate among the cultivars—the number of cultivars (y-axis) in which we observed at least 1 read mapped to the NBS domain (x-axis) in our NBS-capture sequencing data, shown for each of the 587 domains. (**e**) Extent of the NBS domains captured for each cultivar—The number of NBS domains (y-axis) that had at least one read mapped in the NBS-capture sequencing data of each cultivar (x-axis), shown for each cultivar.

The number of reads sequenced for each of the 96 libraries (representing 91 cultivars and breeding clones, as well as 5 pools of genetically closely related individuals of mapping populations) ranged from 1.52 million (M) to 136.81 M, with an average of 15.12 M reads per sample. In total, 1.45 billion reads were obtained (Fig. 2a), of which 84% were mapped (see “Materials and methods”) to the DM v 4.03 genome (^15^, https://solanaceae.plantbiology.msu.edu/pgsc_download.shtml). The mapping rate in the 96 different samples ranged from a minimum of 64.3% (cultivar Satina) to a maximum of 94.6% (Reichskanzler). The complete data is available at the European Nucleotide Archive ENA (accession no. ERP086266).

#### Specificity of the NBS-capture protocol

In total, 296,002,009 sequenced reads (20.4%) mapped to our annotated NBS domains (Fig. 2a checkered area). Although a larger percentage of reads (64.1%) mapped outside of NBS domains, we observed clear enrichment of length normalized read coverage in R genes [number of genes: 704 (703 with coverage in at least 1 cultivar), average gene length: 3,517.91, length range: 302–11,894] as compared to non-R genes [number of genes: 36,952 (36,951 with coverage in at least 1 cultivar), average gene length: 3,062.92, length range: 301–56,876] (Fig. 2b), confirming the specificity of the NBS-capture protocol. Within R genes, NBS domains had substantially higher length normalized read coverage than the non-NBS domains. The non-NBS domains did have some marginal coverage as compared to non-R genes, indicating that short stretches of the adjacent LRR domain were also sequenced. When we evaluated the fraction of bases with read coverage in the different regions, we observed substantially higher sequenced fractions in NBS domains of R genes (Fig. 2c).

#### Sequence read coverage statistics in NBS domains

Details on the NBS domains detected per cultivar can be seen in the Supplementary Table S1. The average length of the continuous portions (overlapping reads) of the NBS domains sequenced by us was 396 bp while the average length of the entire annotated NBS domain per gene was 824 bp. Nonetheless, multiple portions per domain were often sequenced (the Supplementary Table S1) and thus, the average fraction of the NBS domain that was sequenced was 0.86 (Fig. 2c). The sequenced portions of NBS domains had an average of 275-fold coverage per bp. Although most domains were covered by a substantial number of reads, there was variation in the read coverage between the 587 NBS domains. The number of mapped reads per domain, averaged among the 96 libraries, ranged from 0.006 reads per bp in the NBS domain of RDC0001NLR0240 to 96.394 reads per bp in the NBS domain of PGSC0003DMG400029505. The low number of reads obtained for RDC0001NLR0240, a gene that is 3,270 bp long, could be due to the fact that it has a partial NBS domain of length 155 bp on the DM reference genome, i.e., it covers one-fifth of a full NBS domain.

#### NBS domain detection rate among the cultivars

All 587 NBS domains (576 listed in Jupe et al.^13^ and 11 additional, see section “Additional NBS domains detected”) had mapped reads in at least 70 of the 96 libraries (Fig. 2d). An exception was ST4.03ch10:50,899,413–50,899,568 in gene RDC0001NLR0240, a gene in R gene cluster 71, with read coverage in only 42 of the 96 cultivars.

#### Extent of the NBS domains captured for each cultivar

Most cultivars had almost all NBS domains detected, having at least one sequence read mapped to it (Fig. 2e). On average, 584.9 out of all 587 NBS domains were sequenced per sample. The lowest number of domains, 575, was obtained with cultivar 354.00.5 (a breeding clone from INRA Comité Nord). This could have been due to insufficient sequencing depth as only 1,990,294 reads were sequenced in this library.

#### Additional NBS domains detected

We detected 11 NBS domains not recorded in Jupe et al.^13^, indicated in Supplementary Tables S1 and S2 as NB_GTP_1 through NB_GTP_11 (where NB refers to Nucleotide Binding site and GTP refers to Group Tuberosum Pool) followed by the PGSC gene number. Each of these NB_GTP harboring loci, located in genes not previously designated as resistance genes, was represented by a substantial number of mapped reads that were found to contain NBS domains with our DM-specific NB-ARC HMM search. Further confirmation was obtained with a tblastn search for the P-loop, GLPL, and Kinase-2 motifs (from Jupe et al.^12^) in these regions. To determine if the genes had partial or complete NBS-LRR sequences, mast^24^ was used to detect all NBS-LRR motifs (published in Jupe et al.^12^), and NLR-Parser^25^ was used for classification of the gene. We found only 1 out of the 11 genes to be complete. The remaining 10 were partial NBS-LRR genes. In addition, 8 were classified as CC-NBS-LRR (CNL) proteins (Supplementary Table S2).

### Variation detected in the samples (cultivars)

A total of 1,475,836 polymorphisms relative to the DM genome were detected with Freebayes^26^ in the NBS domains across all 96 samples (see “Materials and methods”). On average, 15,573 polymorphisms per sample were counted. Breeding clone 354.00.5 (INRA, Comité Nord) had with 7,326 the lowest number of polymorphisms, while Coquine (Grocep) had with 20,326 the highest number of polymorphisms. This appears to be associated with the total sequencing depth, as was also seen with the total number of detected loci.

**Figure 3:**
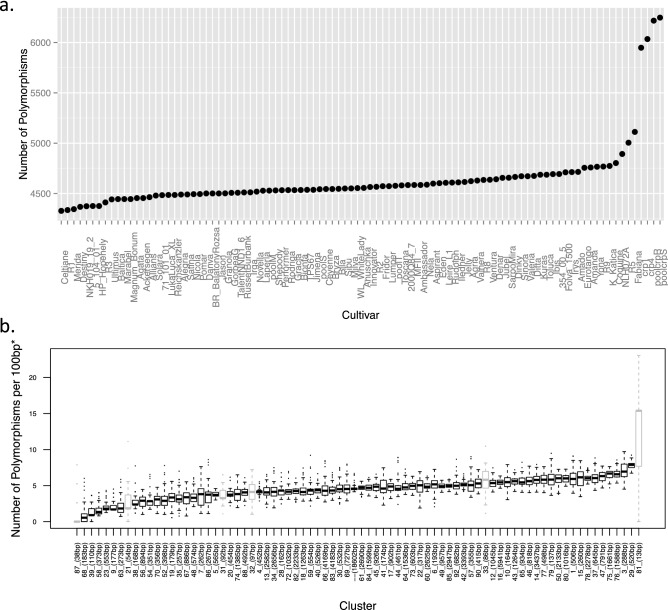
NBS domain sequence variation. (**a**) The number of polymorphisms (y-axis) detected among the cultivars (x-axis) at sites within NBS domains that had at least ×10 coverage in all of the 96 cultivars analyzed. (**b**) The distributions of number of polymorphisms per 100 bp (y-axis), at positions within NBS domains that had at least tenfold coverage in all libraries, over the different NBS-LRR R gene clusters (x-axis) for the 96 libraries. *Lengths of each cluster (shown in brackets in the x-axis labels) were computed by the sum, for all R genes assigned to the cluster, of the lengths of regions within NBS domains that had at least ×10 coverage in all of the 96 cultivars. Clusters with less than 100 bp of at least ×10 coverage in NBS domains of all cultivars have boxplots shaded grey, as the short stretches evaluated in this comparison are unlikely to capture the true extent of the number of polymorphisms in these regions.

For a comparison of the extent of polymorphisms present across all cultivars, without the influence of the effects of sequencing depth, a list of sites within NBS domains that had at least tenfold coverage in all 96 libraries (i.e., no library possesses < 10 sequence reads at the genomic position) was used (Fig. 3a). The average number of NBS domain polymorphisms per cultivar was 4,637. The cultivar with the least number of polymorphisms was Celtiane with 4,328 while the sample with the highest number of polymorphisms was Crp_pool_S with 6,247. The number of polymorphisms among the tetraploid potato cultivars ranged from 4,328 to 5,100 and did not differ substantially from the average. The wild diploid C1 and K4 and pools of their cross progenies, Crp_pool_R and S, had a noticeably higher number of polymorphisms detected (5,948–6,247).

Figure 3b shows the distributions of number of polymorphisms per 100 bp (at positions within NBS domains that had at least tenfold coverage in all libraries) over the different NBS-LRR R gene clusters for the 96 libraries (We used the numbering of R gene clusters according to Table S2a in Jupe et al.^13^). Details on each cluster’s number of NBS domains, total NBS domain lengths as well as the average number of polymorphisms per 100 bp, can be found in the Supplementary Table S3. NBS domains of genes that do not belong to a cluster, singletons, (“–” in Fig. 3b) had an average among the 96 libraries of 4.67 polymorphisms per 100 bp. This is close to the average of 4.53 for all the positions evaluated. Among the clusters with at least 100 bp of at least tenfold coverage in the NBS domains for all libraries (Fig. 3b, black boxplots), cluster 29 had the highest number of polymorphisms with an average among the 96 libraries of 7.83 while cluster 68 had the lowest with an average of 0.74 polymorphisms. The clusters with less than 100 bp lengths of at least tenfold coverage in the NBS domains for all libraries are unlikely to capture the true extent of the number of polymorphisms in these regions and were not considered (Fig. 3b, grey boxplots).

### SolariX website

**Figure 4:**
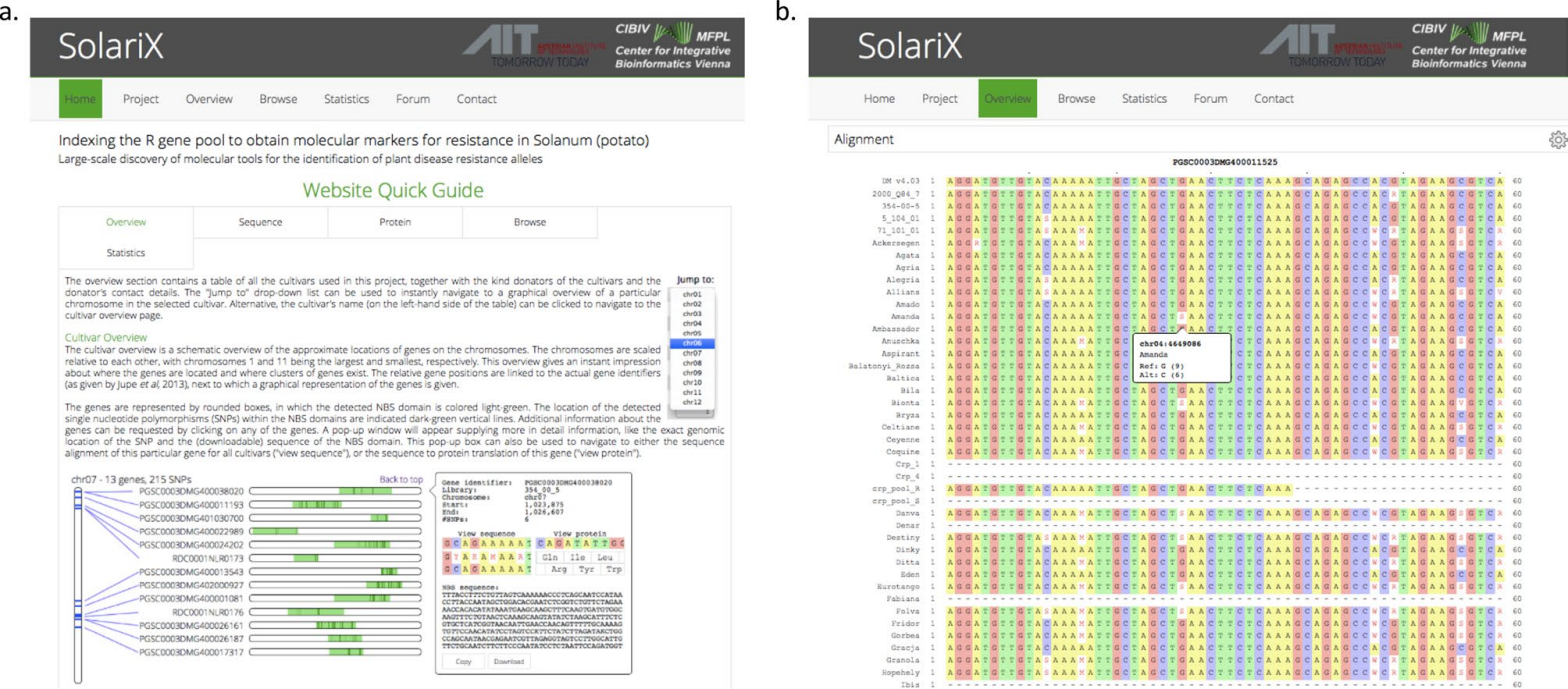
The SolariX website at www.cibiv.at/SolariX provides information obtained from sequencing the NBS domains of 96 cultivars or samples. (**a**) Screenshot of the Quick Guide to navigating the SolariX website. (**b**) Screenshot of the IUPAC sequence per cultivar at the NBS domain of the resistance gene PGSC0003DMG400011525. Clicking on a variant provides read coverage support for each nucleotide of this sequence tag.

The comprehensive resource of variants detected in the 96 cultivars within the NBS domains has been made available on the SolariX website at www.cibiv.at/SolariX (Fig. 4). After registration, one may download the IUPAC code DNA sequences representing the variants found in a cultivar of interest for any annotated NBS domain (Fig. 4a). In addition, polymorphic sites in NBS domains can be visually compared across all 96 cultivars, together with information of read coverage support for each nucleotide (Fig. 4b). We expect that this may facilitate the search for particular resistance alleles in individual cultivars (we include some examples in this manuscript).

### Testing the possibility to detect individual NBS alleles

**Figure 5:**
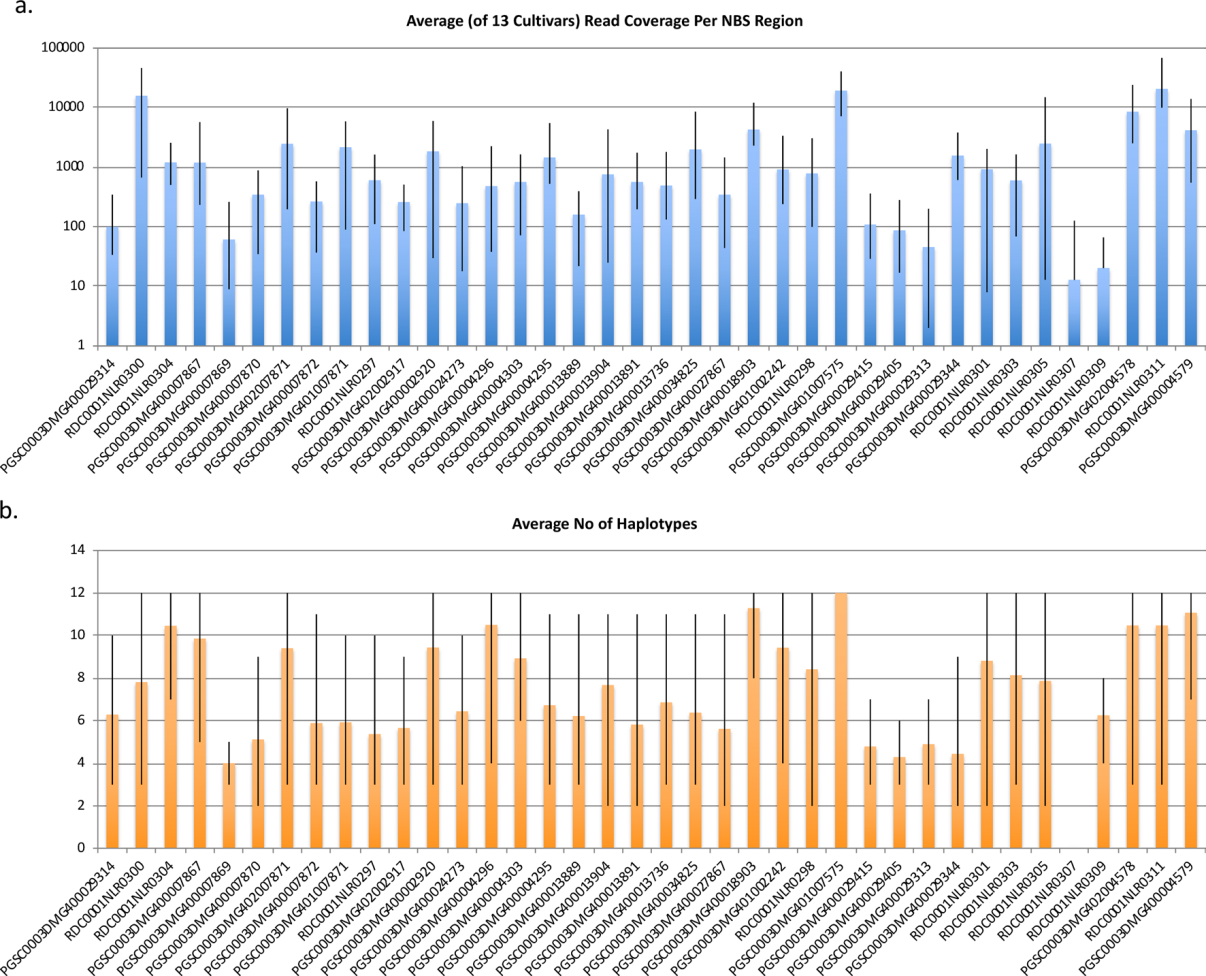
(**a**) Average read count (log scale for y-axis) and (**b**) number of haplotypes phased for 13 cultivars (MFII, TPS67, poolcrpR, poolcrpS, SarpoMira, Alegria, Granola, Russet Burbank, R1, R3, Danva and Balatony Rozsa) at 38 NBS regions on chromosome XII (x-axis). The black 'error' lines represent the minimum and maximum of the 13 cultivars rather than standard deviation.

An attempt was undertaken to identify individual biological alleles (haplotypes) per cultivar. With variant calling using FreeBayes, multi-nucleotide polymorphisms (MNPs) or complex polymorphisms could be identified whenever proximal alleles were on the same read. To extend the analysis beyond individual reads, the variants determined by FreeBayes were used for phasing, i.e. to determine the linkage of variants in assembled reads, within NBS domains using HapCompass^27^. We found that increasing the specified ploidy number beyond 4 resulted in increased haplotypes found. NBS domains of single genes within the genomic library of a single cultivar showed from four up to twelve alleles, thus exceeding the maximum number of four possible in a tetraploid. In Fig. 5, we show the average read coverage (Fig. 5a) and the average number of unique haplotypes found (Fig. 5b) for 13 cultivars at each of the 38 NBS domains on chromosome XII when ploidy was set at 12. No clear trend appears to be present between the read coverage and number of unique haplotypes found.

This made us suspect that we had an issue of "mixture loci", where the regions of interest based on the reference genome actually contained reads from different loci. There were two potential causes of this. Firstly, the DM reference genome differs to an unknown extent from the Group Tuberosum genome of the cultivated varieties we sequenced. It is possible that reads obtained from regions present in the cultivars, but not represented in the reference, map to alternative best hit positions in the DM genome, thus artificially increasing the ploidy at the mapped regions. Secondly, the substantial sequence similarity of the NBS domains across the R genes, a consequence of multiple duplication events in the expansion of NBS-LRR R genes^28, 29^, could cause mis-mapping of reads when sequenced sections of NBS domains are not unique. The issue of read mis-mapping is exacerbated in NBS-LRR clusters with high copy numbers and high sequence similarity. Therefore, it is possible that a locus is assigned reads originating from other loci.

Mis-mapping that results from a combination of high NBS domain sequence similarity and short read lengths (the read length of HiSeq-2000 was 100 bp) could be addressed with longer sequence reads (such as MiSeq; read length, 300 bp), from which sufficiently long sections of NBS domains can be identified, that are distinguishable from other NBS domains of high sequence similarity. Re-sequencing of 6 cultivars or pools of progenies, TPS67, MF-II, poolM, poolT, poolS and clone R2 (late blight resistance differential^9^), was conducted specifically at a region on chromosome IV using a MiSeq sequencer (Illumina) to obtain paired-end reads of 300 bp each. Sequencing depths ranged from 1.18 to 9.47 M. The MiSeq reads were mapped with NextGenMap^30^ at default parameters, as was with our HiSeq reads. This involves local alignment of the read with a required minimum mapped length of 0.5 fraction of the read-length and a required minimum 0.65 fraction identity between the mapped read portion and the DM genome. The mapping criteria are based on fractions of the read length and therefore account for proportional increases in number of polymorphic positions when evaluating longer genomic regions with the MiSeq reads. Despite longer read lengths, the percentage of reads mapped ranged from 60.6% to 64.8%, which were much lower than those obtained in our HiSeq data. We did observe that the sequencing base quality scores at the ends of the MiSeq reads dropped drastically, especially so for the 2nd read of the pair, which could have contributed to the lower mapping rates.

Although we anticipated that longer reads would allow for better distinction between highly similar NBS domains, we found an increase in the percentage of reads that mapped to multiple locations from an average of 22.23% of the mapped reads in the HiSeq libraries of the 6 cultivars to an average of 33.56% of the mapped reads in the MiSeq libraries. In addition, mapped reads having a mapping quality of greater than 20 decreased from an average of 27.03% of the mapped reads in the HiSeq libraries to an average of 10.03% of the mapped reads in the MiSeq libraries. The DM reference represents a rather small section of the whole genetic variability within the cultivated potatoes, and the greater percentage of unmapped reads as well as decrease in mapping qualities in our MiSeq data suggests considerable sequence divergence between the Group Tuberosum cultivars to the reference. Further examination of the unmapped reads, for a single MiSeq library (MF-II), using a blastn search to the NCBI nucleotide database revealed that 62.88% of the 648,960 reads had a significant blast hit (e value smaller than 1e–5) in either the Solanum genus (7.72%) or the tetraploid species *Triticum turgidum* (emmer wheat, 44.24%) to mostly nucleotide sequences representing R genes (Supplementary Figure S1). The unmapped reads therefore could contain novel R gene content that is currently absent from the DM genome. In fact the DM genome remains incomplete, with improvements still ongoing. This provides strong evidence that insufficient representation on the reference genome was a greater issue than short read lengths and high sequence similarity.

### Inferring genetic similarity between cultivars

**Figure 6:**
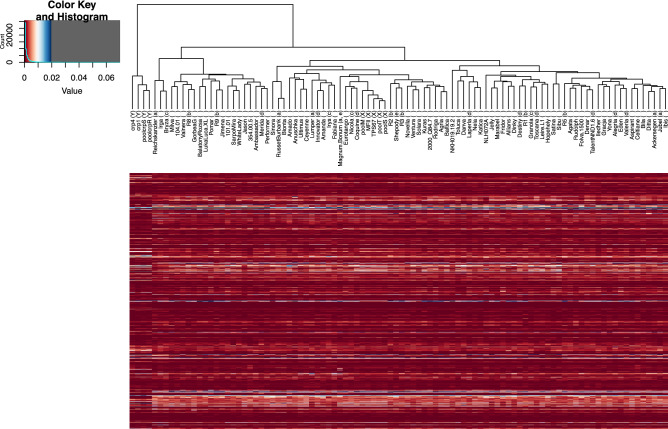
Inferring genetic similarity between cultivars based on variations in read coverage for nucleotide binding site domains. Read coverage frequencies (RCF), by resistance (R) locus on the reference genome DM, of all 96 cultivars represented as a compressed heat map. The sum of RCFs over R loci per cultivar is 1. The dendrogram represents hierarchical clustering based on the Euclidean distance between cultivar RCFs. The color key at the top right corner illustrates the correspondence between RCF value (x-axis) and color. A histogram (y-axis) of RCF values observed in the heatmap (turquoise line) is included in the color key. Cultivars were classified into the following groups (letters in parentheses at the labels of the dendrogram tips) – Five libraries representing parents and pooled progenies of population MT (X); Four libraries of our outgroup taxon, S. caripense, consisting of parents C1 and K4 and two pools of cross progenies (Y); Six individuals registered before 1930 (1810–1929) (a); Six late blight resistance differentials (ca 1950–1970) (b); Four cultivars released 1972–1975 in Germany and Poland (c); Nine contemporary cultivars registered by seven breeders between 1999 and 2011 in four countries (d); Two cultivars of (partially) American descent (e); All other sequenced cultivars ( )

If certain genomic regions in our sequenced varieties lack representation on the DM genome, and reads from these regions map to alternative best hit positions, the variation of coverage of sequence reads obtained in the NBS domain per R gene (read coverage frequency, RCF) may be useful as a measure of divergence of a certain variety to the reference genome, DM. We hypothesized that the percentage at a single domain, out of all reads obtained for a cultivar could be informative, to some extent, about the genetic diversity. This approach implicitly allows us to utilize the copy number variations of R genes within clusters, which we expected to differ even on the cultivar level. Therefore, the absolute numbers of reads at the individual NBS domains of a cultivar were represented as a percentage of the total reads mapped to all NBS domains obtained for this cultivar (i.e. the sum of RCF values of the 587 NBS domains equals 1 in each cultivar library). These normalized figures, we termed NBS domain RCF vectors, were then used to calculate the Euclidean distance between all pairs of cultivars. A dendrogram of hierarchical clustering using the Euclidean distance and a compressed heat map of the read frequencies by NBS domains of R genes of every cultivar is shown in Fig. 6, details can be seen in the Supplementary Fig. S2. The four libraries of our outgroup taxon, *S. caripense*, consisting of parental C1 and K4 and two pools of cross progenies (Group Y in the Figure), form a clearly distinct cluster distant to all potatoes, as was expected. The five libraries representing parents and some progenies of population MT (Group X) also clustered together showing only minimum distances. Overall, all potatoes were distributed across two major branches, one containing 21 cultivars (Fig. 6, Reichskanzler—Performer) and the other 71 (Sinora—Ibis). The first branch containing 21 cultivars shows two distinct sub-branches, one containing Reichskanzler, Irga, Bryza, and the other the remaining 18 cultivars. The second, right-most potato branch in the dendrogram holding 71 cultivars shows two sub-branches that branch off further into several less distant groups. We selected several cultivars whose age, purpose of creation, or country of origin was distinct, to elucidate various attributes of the potato’s pool of R genes. These groups of cultivars (Figs. 6 and S2, Table S4) were ‘a’: 6 individuals registered before 1930 (1,810–1,929), ‘b’: 6 late blight resistance differentials (ca 1950–1970), ‘c’: 4 cultivars released 1972–1975 in Germany and Poland, ‘d’: 9 contemporary cultivars registered by seven breeders between 1999 and 2011 in four countries, and ‘e’: 2 cultivars (Shepody and Magnum Bonum) of (partially) American descent. Magnum Bonum was selected by J. Clarke in the UK from a cross of (the American) Early Rose × Paterson’s Victoria, and Shepody was selected in Canada (www.europotato.org/). As can be seen from Figs. 6 and S2 and Table S4, the old cultivars of group ‘a’ fall into several distant clades indicating their large diversity. An exception are Ackersegen and Jubel at a minor distance, they originate from the same breeding program (Böhm, Germany), although their pedigrees (as indicated in the European Cultivated Potato Database; www.europotato.org/) are quite different.

The peculiar distribution of the late blight differentials (group ‘b’) across the dendrogram seems to reconstruct the lines how these clones were created; R1 was found first, followed by R2 and R3, while R5, R8 and R9 were developed in later years when new sources of late blight resistance had become accessible^9, 31^.

The modern cultivars of groups ‘c’ and ‘d’ mainly do not share the same subgroups in the dendrogram indicating that certain levels of diversity may have been maintained over the generations and programs of potato breeding.

Magnum Bonum and Shepody (group ‘e’, in part American descent), are interspersed among the European cultivars confirming the common origins of these breeding pools. One hundred years lie between the two cultivars—Magnum Bonum was registered in 1876 and Shepody in 1980—but nonetheless, the relative read frequencies at their NBS domains do not seem to divert very much (Fig. 6). Shepody is in a small cluster together with the UK clones R2 and R3, and Magnum Bonum forms the base of a neighboring cluster holding the modern cultivars Eurotango, Nicola, Coquine, MF-II and TPS67. MF-II is of Indian descent, which originated in breeding stocks created by the UK based W. Black and co-workers in the early 1900s and which may therefore share the same specific genetic background.

### Using the SolariX database to efficiently discover R alleles responsible for specific resistances: can it be used to create selection markers for these alleles?

In an approach alternative to the detection of biological alleles (which was not unambiguously possible), we continued to base our analyses on individual polymorphisms and searched for candidate SNPs indicative of the specific disease resistances present in our experimental plant materials. For the two groups of libraries M, T, poolM, poolT, poolS and C1, K4, Crp_pool_R, and Crp_pool_S, we searched for polymorphic nucleotides that were in differential states within the disease resistant vs. susceptible parents and pools of progenies.

#### Development of molecular markers for a late blight resistance gene from S. caripense (CRP)

The late blight resistance originating in the parental genotype C1 was previously mapped in the CK population^23^ to the CRP homolog of potato chromosome IX. All polymorphic positions on chromosome IX from the Illumina HiSeq sequence data were therefore analyzed for the conditions that the resistant parent C1 and the pool of resistant progenies Crp_pool_R had a shared SNP allele and that this allele was absent from the susceptible parent and pool of susceptible progenies. Such SNPs should be covered by a minimum of 10 mapped reads, and the numbers of reads covering the reference and alternative allele detected should fit the expected ratios (tested by *chi*-square tests for goodness-of-fit) according to the genetic model applied. A series of 46 SNP sites fulfilling these conditions were detected, exclusively across the genome region holding the R gene clusters 63 to 66. Seven of these candidate SNPs occurred within a narrow section of 445 bp at chromosomal positions 59,553,212 to 59,553,656 bp within the NBS domain of R gene PGSC0003DMG402020585 (cluster 64). This was the biggest collection of intra-gene occurrence of putative resistance-associated polymorphisms and therefore, we selected it as a promising candidate and designed locus-specific PCR primers for amplification of a fragment to cover most of the P-loop, Kinase-2 and GLPL motifs. The genomic positions of the primers were 59,553,177 (primer 2020585_F; TGTTCCGTTCTTTCTCTTGC) and 59,553,855 bp (primer 2020585_R; CATTCCATTGGACCAGAGAG) on DM v 4.03 chromosome IX. The sole 696-bp PCR product obtained with these primers was treated with several restrictases in order to detect SNP variants corresponding to the haplotype of the (late blight resistant) parent C1 and Crp_pool_R group of progenies, according to the Illumina sequencing data (compare Supplementary Methods S1).

#### Choice of a SNP and development of a molecular marker for resistance originating in parent C1

Information obtained upon digestion with two restriction enzymes was useable to distinguish resistant and susceptible progenies in the CK population. The resistant parent C1 was heterozygous at a single nucleotide positioned at 59,553,259 bp (relative to the DM genome) at a locus within R gene cluster 64. Paradoxically, all late blight susceptible progenies shared a cutting site for *Tai*I (Thermo Fischer) with the resistant parent C1, neither the susceptible parent K4 nor any of the resistant progenies showed this feature. After digestion of the same PCR product with *Dde*I (New England Biolabs) targeting a neighboring SNP at position 59,553,275 bp, this digestion pattern was observed again suggesting that the two CAPS (Cleaved Amplified Polymorphic Site) markers originate in the same haplotype. Therefore, visualization of both *Tai*I or *Dde*I digestion products of CRP DNA amplified by the aforementioned primer pair can serve as ad hoc CAPS markers for the selection of resistant and susceptible progenies from this genetic background. Progeny giving a PCR product that is cut neither by *Tai*I nor *Dde*I are likely to be late blight resistant.

#### Verification by Sanger sequencing

PCR with the above described primers was applied on template genomic DNA of the parents, the two pools, R and S, and an additional 71 individual CK progenies of our mapping CRP population (30 possessing a resistant and 41 a susceptible phenotype as observed in multiple bioassays with virulent isolates of *P*. *infestans*, from Nakitandwe et al.^23^). The fragments were subjected to Sanger sequencing (LGC Genomics, Berlin, Germany) and yielded analyzable 596-bp sequences. All fragments shared the same sequence containing 15 polymorphic single nucleotides that segregated in association with the resistance phenotype. The pattern of these SNPs relative to the status of late blight resistance of the individual parents and progenies indicated good but not perfect association (Table 3). Frequently, the resistant C1 was heterozygous and the susceptible K4 homozygous, whereas this pattern was unexpectedly reversed in the progenies, the resistant offspring having the homozygous haplotype corresponding to the susceptible parent. One exception was the SNP at the fragment’s position 397. Parent C1 had genotype C, the pool of resistant progenies and all 30 resistant CK individuals tested were heterozygous CT, whereas parent K4, Crp_pool_S and all 41 susceptible CK progenies were homozygous T. Hence, even for this SNP the peculiar genotype of C1 (homozygous C instead of an expected heterozygous CT) was not in accordance with the suggested by the 1:1 segregation of all cross progeny for resistant/susceptible phenotype. Moreover, the SNPs detected on the Illumina sequence data differed from those of the Sanger data (Table 3).

**Table 3:** SNPs showing patterns proportional to late blight resistance in *S. caripense* (CRP).

SNP position^†^	C1^‡^	K4	PoolR	PoolS	Resistant progeny	Susceptible progeny	# No match^§^	Chromosome IX; 15,686,521 bp^¶^
								Locus PGSC0003DMG400004413 Illumina: 37 SNPs
004	C/T	T	T	C/T	T	C/T	13	004; C, T
100	A/T	A	A/T	A/T	A/T	A	14	095; A, G, T
109	A/T	T	T	A/T	T	A/T	7	–
198	A/G	A	A/G	A/G	A	A/G	6	197; A, G
237	C/T	C	C	C/T	C	C/T	6	–
259	A/G	G	G	A/G	G	A/G	5	–
275	C/G	G	G	C/G	G	C/G	6	–
296	C/T	T	T	C/T	T	C/T	5	294; T, C
301	C/T	C	C	C/T	C	C/T	6	302; A, G
355	A/G	A	A	A/G	A	A/G	7	349; C, T
370	C/T	T	C	C/T	C	C/T	5	369; C, T
397	C	T	C/T	C	C/T	C	4	395; C, T
408	A/G	A	A	A/G	A	A/G	5	403; A, G
421	A/G	A	A	A/G	A	A/G	6	421; C, G
503	A/G	G	G	A/G	G	A/G	6	–

Test for association of the late blight resistance phenotype (as determined in multiple bioassays by Nakitandwe et al.^23^) with SNP haplotypes on PCR fragments of genomic DNA amplified using site-specific PCR primers on CRP parents (C1; heterozygous for dominant late blight resistance, K4; susceptible), pools representing 10 resistant or 10 susceptible progenies, and 71 individual progenies (30 late blight resistant, 41 susceptible). The PCR primers pinpoint a conserved stretch of R gene locus PGSC0003DMG402020585. The fragment was Sanger-sequenced. The first nucleotide of this locus (position on fragment, 001) maps to position 59,553,199 bp on potato chromosome IX. The locus is a member of R gene cluster 64 which also holds a late blight resistance gene isolated from *S. venturii*^53^.

^†^SNP as observed on Sanger-sequenced fragments PCR-amplified from C1, K4, and CK progenies, ‘Pool_R’ and ‘Pool_S’; pooled DNA samples of 10 resistant or 10 susceptible CK progeny individuals that were randomly chosen, Resistant progeny, Susceptible progeny; total of 30 individual CK segregants possessing a late blight resistant phenotype and 41 susceptible segregants, respectively.

^‡^C1; late blight resistant parent (K4; susceptible parent) of CK population (cross of C1 and K4, *S. caripense*, 2n = 2x = 24),

^§^number of individual samples tested (in total 75) where resistance phenotype and SNP genotype were in disaccord with the rule (e.g., genotype C/T when the rule was T for resistant individuals among the progenies),

^¶^position of SNP as observed on the Illumina Hiseq-2000 data (closest to those detected on Sanger-sequenced fragments, left columns). For the R gene locus on chromosome IX shown here, the best coincidence of SNPs as found on Sanger sequenced fragments out of nine loci investigated (see Results) was obtained. However, this locus is distant from the region where the resistance was mapped (Nakitandwe et al.^23^).

We aligned all fragments whose sequence was determined by the Sanger method via a blastn search onto the DM v 4.03 genome. This returned nine separate R gene loci on chromosome IX with (considerable) sequence similarity of 88 to 92% along uninterrupted stretches of 473–593 bp of our fragments. Therefore, we examined all nine regions for the occurrence of SNP positions in the mapped NBS-tags of C1, K4, Crp_pool_R and Crp_pool_S. This was done by comparisons of the 15 individual SNP positions on our Sanger-sequenced fragments with SNP positions as detected on the mass sequencing data. Unfortunately, none of the nine candidate sites on the genome models perfectly matched the structural patterns of the SNP positions as detected by Sanger-sequencing (Table 3, rightmost column).

### Search for markers of two R genes conferring resistance to late blight in the MT population

The Illumina sequences obtained for the five libraries of the MT population parents and three groups of MT progenies representing distinct resistance phenotypes were subjected to the same approach used for population CK data (described above). Two unknown major dominant genes for resistance to late blight segregate in the MT population, one originates in MF-II, chromosome XI, and the other in TPS67, chromosome IV^32^ and their phenotype of resistance can be distinguished upon the interaction with differential isolates of *P. infestans*. Many clustered SNPs within NBS domains of R genes on chromosomes IV and XI were detected. The largest number of clustered SNPs displaying the segregation pattern expected for a dominant R gene originating in TPS67 was found in several NBS domains on cluster 16, which also holds the closest DM sequence relative to the *R2* late blight resistance gene of the Black et al.^9^ series. As an example, seven adjacent SNPs were found in the NBS domain of PGSC0003DMG400011525 that display the haplotypes MF-II; 0, TPS67; 0, 1, poolM; 0, poolT; 0, 1, and poolS; 0 (where 0 represents the reference nucleotide/allele of the DM genome and 1 represents an alternative nucleotide) and hence suggested the segregation of a single factor (denominated ‘1’) from parent TPS67 in accordance with the phenotype of resistance. A total of 86 SNPs across 45 NBS domains of R genes in 13 clusters on chromosome IV displaying this pattern of segregation and potential association with the resistance gene of this parent were detected. Cluster 16, the cluster of NBS-LLR R genes that harbors the original gene *R2*, alone had 36 SNPs across 18 NBS domains, one of these within PGSC0003DMG400032572, the closest DM sequence relative to the *R2* gene. Likewise for chromosome XI, 171 clustered SNPs across 61 NBS domains of R genes in 13 clusters or singleton NBS R genes were found in the NBS-capture data that suggested co-segregation with a resistance gene from MF-II. The experimental elaboration of these candidate SNPs and loci is in progress.

#### Development of molecular markers for a PVY (Potato Virus Y) resistance gene

**Figure 7:**
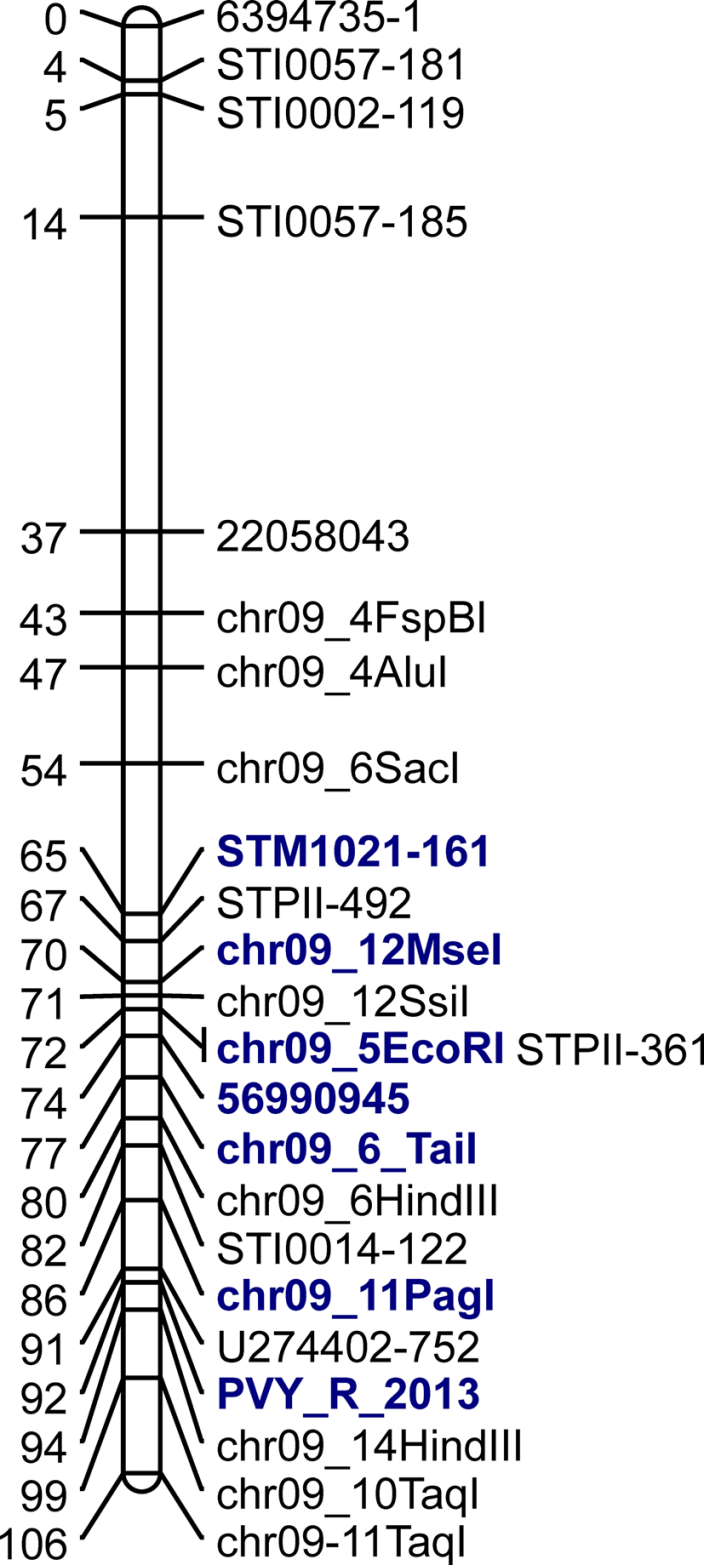
Genetic map of Alegria chromosome IX. Right; relative molecular marker positions, left; genetic distance (cM). Markers in bold print are linked in coupling phase with the PVY resistance.

An in-depth analysis was conducted for the parents of the AB mapping population comprising 250 individuals from the cross: Alegria x Baltica. Cultivar Alegria inherits resistance to PVY in a qualitative, dominant fashion suggesting the action of a single R gene, and selection for this resistance with molecular markers could thus facilitate breeding for PVY resistance. From framework genetic mapping (Supplementary Methods S2), the PVY resistance was found to be in linkage with all four chromosome IX markers used; STI057, STI002, STI014^33^ and STM1021^34^ and not with any marker on any other chromosome (Fig. 7). Hence, we examined our Illumina sequence data of both parental cultivars, Alegria and Baltica, for polymorphisms on chromosome IX that could segregate in association with the PVY resistance of Alegria. We expected the Alegria PVY resistance allele to be in simplex state, according to the 1:1 segregation pattern of resistant and susceptible progenies in the AB population. We looked for polymorphic sites within every NBS domain that were covered by at least 20 reads and that contained SNP alleles that occurred in parent Alegria at a frequency of 0.25 (verified by a *Chi*-square test) but were absent in Baltica. PCR primers (containing a mismatch nucleotide at position 3′-1, see “Materials and methods”) for four of the polymorphic sites that corresponded to these conditions were designed and applied on the parental and progeny DNA. The results were integrated into the genetic map of cv. Alegria. With these additional markers, it was possible to assess the position of the PVY resistance to be in the vicinity of, or within clusters 64 (containing the prominent locus *Tm-2* for tomato mosaic virus resistance) to 66 (holding the potato homologous locus of tomato *Sw-5* for resistance to tomato spotted wilt virus). Consequently, the specific NBS domains of the tomato *Tm-2* and *Sw-5* homologous potato loci in the DM v 4.03 reference genome were used as models to design 14 PCR primers for the selective amplification of these specific NBS domains (Supplementary Table S5) on the map of chromosome IX enriched with additional markers (Supplementary Tables S6 and S7). These primers were applied on Alegria and Baltica DNA and the fragments amplified were cloned and subjected to Sanger sequencing. The resulting 349 sequence reads were aligned with NextGenMap at default parameters to the DM v 4.03 to verify their exact position on chromosome IX and to search for polymorphic nucleotides of Alegria relative to Baltica. All Sanger reads mapped to chromosome IX of the DM reference. 109 out of 173 and 111 out of 176 reads from Alegria and Baltica respectively mapped within R genes of clusters 64–66. When comparing regions of the genome where both Alegria and Baltica Sanger reads mapped, we found 190 polymorphic positions where Alegria reads contained a SNP allele, with at least 2 supporting reads, that was not observed in the Baltica reads. 15 were within R gene singletons, 32 were not within R genes and 143 were in R genes from clusters 64 to 66. Seven of the 14 original PCR primers targeted SNPs with a predicted restriction site (compare Supplementary Table S5; primers with restriction enzyme information). We applied these primers to the parents and all progenies of the AB population and treated the amplicons with site-specific restrictases to search for informative CAPS markers. All seven primers produced segregating markers and we included these into the genetic map of Alegria. Among them, the CAPS marker chr09_11*Pag*I was of all markers closest to the map position of the PVY resistance (Fig. 7). This marker originated in cv. Alegria and was in simplex state and in coupling phase, 6 cM away from the putative, simplex PVY resistance. The marker is located in RDC0001NLR0230 of cluster 66. The map location of the PVY resistance was deduced from the performance of all individual AB progenies in replicated resistance trials. The semi-quantitative nature of these trials rendered the interpretation of results regarding the presence or absence of a single, qualitative resistance factor somewhat dependable. Therefore, chr09_11*Pag*I was proposed as a marker to select for the PVY resistance inherited by cv. Alegria.

## Discussion

### Possibility to detect and count NBS-LRR haplotypes using NBS locus tags

We were able to detect NBS domains of up to 587 R genes using selective amplification by PCR and high-throughput sequencing. Thus, a total of just 16 primers were sufficient to develop a comprehensive inventory of virtually all NBS domains of the NBS-LRR R genes in the DM genome. Altogether, three hundred million reads were obtained from 91 distinct cultivars and were mapped to 587 NBS domains of the reference genome. The amount of information that can be currently drawn from this data, however, is limited due to apparent mis-mapping and consequently, intermixing of the individual fragments to an unknown extent. This was evident from the numbers of haplotypes inferred for the NBS domains evaluated that frequently exceeded the maximum of four individual haplotypes (alleles) for a single domain and cultivar. A likely source for the multiple haplotypes observed per domain is the copy number variations of R genes in clusters that can differ even at accession level^21^. As proposed by Michelmore and Meyers^35^, tandemly repeated genes within a cluster are unstable because of a high degree of sequence similarity resulting in relatively frequent unequal crossing-over. This leads to further duplications and deletions. The observed differences in read mapping percentages are likely due to the varying divergences between the sequenced cultivar or sample and the reference DM genome. Correct mapping of R gene fragments (that are largely similar due to the high levels of sequence conservation among their NBS domains) to a genome that may be considerable different remains challenging. Our attempt to increase the level of individuality of sequenced fragments via obtaining fragments that include larger parts of variable, non-conserved sequence of the target genes and generating longer sequence reads proved to be ineffective. Thus, the most important means for achieving accurate allocation of sequence fragments should be the provision of high-quality reference genome(s) of the common potato, *S*. *tuberosum* Group Tuberosum.

### What are the numbers of potato NBS-LRR R genes?

Improvements to the potato’s reference genome, based on the whole genome sequence of *S. tuberosum* Group Phureja doubled monoploid clone DM1-3 516 R44 are still ongoing. Our work began on DM version 3.4 although we continued and based our final analyses on v 4.03, and an advanced version 4.04 has been issued in 2016 (https://solanaceae.plantbiology.msu.edu/). The detection and annotation of NBS-LRR R genes has evolved in parallel. Jupe et al. (2012)^12^ counted 438 such loci for DM v 3.4 which we used for the design of our NBS-capturing PCR primers. Jupe et al.^13^ increased the number of NBS-LRR R genes of the potato DM genome to a total of 755, of which 704 were allocated to positions on the twelve chromosomes. The existence of NBS domain-associated motifs is a precondition for our method of detecting such loci by PCR with primers targeting the NBS domain. We detected in our study 576 of the 704 NBS-LRR loci established by Jupe et al. (2013). Although we did not detect an NBS domain in 128 of the 704 R genes, 703 genes had read coverage in at least one cultivar, indicating that they were not completely absent from all our 96 genome libraries. Additionally, we found 11 previously unestablished regions on the DM genome containing an NBS domain. Thus, the number of putative NBS-LRR R genes on the 12 chromosome models of DM v 4.03 has been increased by 11 and the number of NBS domains detected by our NB-tagging method was 587. The high level of sequence similarity among the NBS-LRR domains was a challenge in our project. Our approach was based on mapping of the individual reads to a reference genome, DM v 4.03. Mis-mapping of reads can occur when the reference is too different from the genome that was sequenced. Reads that originate from genomic sequences that are not represented on the reference are incorrectly mapped to the next most similar location in the reference. In addition, ambiguity in mapping comes about from multiple equally divergent (with respect to the read being mapped) locations on the reference, which render the distinction of the true location impossible. We believe the DM v 4.03 reference genome may not adequately represent the genomes of our cultivars and this is based on three observations—First, DM is a (mono) haploid of a genotype that has a rather limited allelic diversity and it is derived from potato Group Phureja which may not share all features with Group Tuberosum genotypes. Second, we observed that on average 23% of the mapped reads were not properly paired; this were pairs that, when mapped on the genome, either had an incorrect orientation, were not within a reasonable distance or the pair members even mapped to separate chromosomes. Thirdly, libraries sequenced with 300 bp paired-end reads (obtained on an Illumina MiSeq) showed substantially lower mapping rates and confidence (mapping quality scores) than the libraries with 100 bp paired-end reads (sequenced on a HiSeq-2000). The unmapped reads were found to have significant best blastn hits in resistance genes in the *Solanum* genus and the wheat *Triticum turgidum*. This suggests that novel R gene content is currently absent from the DM genome.

### Genetic similarity between cultivars

A relatively useful approach presented in Fig. 3a, where the total numbers of polymorphic sites are compared among all 96 DNA libraries, provides a measure of estimating genetic similarity. Unrelated sequence fragments, although sharing roughly similar sequences, will have polymorphic sites at different positions. When fewer non-related sequence reads are present (for example in an inbred, less heterozygous genotype derived from a narrow genepool), fewer such reads can be accidentally assigned together on the same loci and the resulting number of polymorphic sites tends to be reduced. The group of seven libraries showing the smallest numbers of polymorphisms and hence indirectly presenting reduced genetic variability (Fig. 1c, left tail, 4,328–4,376 SNPs per library) contains four modern cultivars (registered between 1997 and 2011), two concurrent (after 2010) breeding clones and only one historical late blight differential clone (R1, published in 1953^9^). Today, potato breeding still depends on pedigree schemes where a relatively small group of elite cultivars are intercrossed. The continued repetition of this scheme over decades, where the genomes of the same parents and grandparents are repeatedly reshuffled, might lead to an enrichment of advantageous alleles while undesired alleles are steadily selected against, thereby leading to an overall reduction of genetic variability.

Another approach at indirectly estimating the genetic variability was the analysis of Illumina read coverage frequencies (RCF) per individual NBS domain of an R gene across the genome libraries (Figs. 6 and S2). This approach allows us to utilize the copy number variations of R genes within clusters. RCF (a numerical criterion) is certainly less precise for assessing the extent of genetic variability of R genes within a genepool compared with the frequencies of true biological alleles. However, the genetically closely related individuals in our data set (the CRP complex and the MT parents and progenies, respectively) clustered closely together by their RCF patterns. CRP, the outgroup species distant to the potato, formed a separate group by its RCF features (Figs. 6 and S2), similar to its distinct level of polymorphic sites (Fig. 1c). MF-II, a clone of Indian descent, and TPS67, Neotuberosum, are within the same narrow group of 20 cultivars (including Magnum Bonum, registered in the UK in 1876, to Baltica, Germany, 1997, and three pools of MT progenies, Figs. 6 and S2), this underlines further, the relative narrowness of the Group Tuberosum R genepool. The continued introduction of individual R alleles and additional R loci (compare *R1*,^10^) did not change the frequencies of particular features within the genetic code of NBS R genes. The Irish cultivar Lumper that survived the 1845–47 late blight calamity, which wiped out significant parts of the early potato’s phenotypic diversity shares the frequencies of major conserved R gene motifs with Innovator (bred at HZPC, NL, registered in 1999), Amanda (Solana, DE, 2006), Irys (IHAR, PL, 1975) or Fabiana (Germicopa, FR, 2008). Therefore, we postulate the potato’s NBS R gene family remained relatively homogeneous over two centuries throughout a wide range of cultivars from many countries and breeders. This is consistent with findings of high synteny, even on the genus level, of the sub-families of NB-LRR R genes between the genomes of tomato (*Solanum lycopersicum*) Heinz 1706 and the DM potato reference, with differences only in smaller cluster sizes in the tomato^36^. The presence of self-incompatibility and the polyploid nature may have supported the maintenance of the common potato’s genetic plasticity. Genetic variation underlying specific disease resistance traits concerning individual biological alleles at individual R loci, such as those comparatively few that have been introgressed from wild and native cultivated sources over the past one hundred years, could not be made visible with the NBS tagging method presented.

### Using the SolariX database to create selection markers for R alleles responsible for specific resistances

We tested two approaches—in the first, SNPs associated with a resistance were determined by matching expected ratios for allele frequencies in both parents and progenies within mapping populations (CK, MT) and in the second, potential SNPs within the region where a resistance maps to were determined by allele frequencies solely in the parents (population AB).

For the CK population of CRP segregating for a single late blight resistance factor, we found markers that have considerable predictive power to assess a progeny’s resistance status. However, the individual locus and allele encoding the late blight resistance could not be detected. CRP is a relative of the potato and tomato, it has a larger genome size (1.2 billion bp, as determined by flow cytometry) and it may have structural differences. Therefore, mapping the NBS-LRR gene fragments from CRP to the DM genome, based on best similarity fit, would inevitably produce inaccuracies. Information on the haplotype responsible for the resistance is certainly intermixed with information on other, unrelated loci that share similarity by their sequence.

For the potato population MT (such as for CK) segregating for two independent, dominant late blight resistance factors, we detected many loci at which groups of SNPs indicated potential co-segregation with the resistance phenotype, based on their allele frequencies. This was not unexpected, as the two parental genotypes M and T (such as parents C1 and K4 of the CK population) should possess many more distinct R alleles than merely the two responsible for their resistance to late blight. The challenge is to precisely locate those polymorphisms that are truly associated with the late blight resistance. This may be achieved by designing PCR primers specific for every individual SNP and testing MT parents and progenies for the presence or absence of markers or by fine-mapping the resistance using many more MT progenies.

Further refinement of our method to combine high-throughput sequencing of longer (several thousand instead of several hundred nucleotides) DNA fragments generated with targeted amplification to capture the true biological alleles of the NBS-LRR R genes with mapping to advanced versions of appropriate reference genomes or, alternatively, reference genome-independent methods, as has been done for an R gene in the wild diploid potato relative *Solanum americanum*^37^, might however provide more straightforward ways to investigate the diversity of plant resistance genes.

The methodology to retrieve molecular markers for the PVY resistance segregating in the AB population differed from the CK method described above inasmuch as it relied on direct information about individual SNP sites retrieved from the Illumina reads of this population’s parents, cultivars Alegria and Baltica. In a search that involved both AB parental information and the DM reference genome, individual SNPs were found that could be used to design CAPS markers for selection of resistant segregants. Again, the markers obtained were linked to, but not directly located within, the responsible putative R locus. This apparent imprecise targeting of the resistance locus was owed to the high redundancy of the NBS R genes in the potato genome and to the dependence of the method on the DM reference genome.

## Conclusion

The SolariX compendium (https://www.cibiv.at/SolariX/, European Nucleotide Archive Study ID PRJEB83917) provides a useful resource of the common potato’s R genepool, for nearly all NBS domains on the DM genome. The presented variability may exceed the true genetic diversity due to inadvertent mis-mapping of highly homogeneous NBS tag reads because of the extreme levels of NBS sequence similarity and insufficient representation on the DM genome. For high-resolution analysis of individual cultivar-specific genomes more expensive methods such as SMRT RenSeq^37^ may be appropriate.

Molecular markers for selecting specific resistances can be developed based on the SolariX database if methods allowing for more precise results are less economical or unavailable.

## Materials and methods

### Plant materials

Eighteen European potato breeding institutions and the GLKS gene bank at the Leibniz Institute of Plant Genetics and Crop Plant Research (IPK), Germany, kindly provided tuber or leaf samples. In total 96 samples consisting of 6 historical and 69 contemporary potato cultivars, 8 breeding clones, 6 R gene differentials for the detection of specific races of *Phytophthora infestans*, the causal agent of late blight^9, 31^, 2 parental accessions of the wild species *S. caripense* (H. & B. ex Dun.), here referred to as CRP, and 5 pools of progenies from two mapping populations representing distinct classes of disease resistance were selected for the experiment (Supplementary Table S4). The materials represent up to 26 European and 3 American, original as well as present-day, potato breeders and their corresponding (sometimes interrelated) genetic stocks that have been developed across 10 European and 2 North American countries since the second half of the nineteenth century until present. The mapping populations of biparental crosses were created to study, in separate, the genetic source of resistance to *Potato Virus Y* (PVY); population AB (a cross of cultivars Alegria and Baltica) and two sources of resistance to late blight; population MT (a cross of tetraploid cultivars MF-II and TPS67, a representative of the Neotuberosum developments; Plaisted 1987) and population CK (diploid *S. caripense*; C1 x K4). Population AB samples and results on virus resistance were provided by NORIKA, Germany. Population MT^22, 32, 38^ is a cross of potato groups Tuberosum and Neotuberosum^39–41^ segregating for two different major, dominant factors of resistance to late blight. Population CK^23, 42^ resulted from a cross of a late blight-resistant and a susceptible individual of the wild, diploid, non-tuberous CRP, a distant relative of the potato and tomato.

### Design of primers for amplification of R gene-specific sequence tags for Illumina sequencing

Primers were designed for three conserved motifs of the NBS domain of major R genes namely, P-loop, Kinase-2 and GLPL^43, 44^ present within all 438 NBS-LRR R gene sequences in the potato DM v 3.4 reference genome as reported in Jupe et al.^12^. The sequences of these three NBS motifs were grouped by similarity across all 438 loci. For each high-similarity group, specific degenerate primers were designed. Thus, according to their ability to amplify from genomic DNA in tests, a total of fifteen primer pairs, seven specific for the P-loop, three for the Kinase-2, and five for the GLPL motifs (Table 1), and in addition the NBS5 primer from Van der Linden et al.^16^, were finally chosen for our PCR enrichment of NBS containing genomic fragments across all 96 varietal DNA libraries (Table 1).

### Isolation of R gene fragments for Illumina HiSeq-2000 sequencing and profiling of NBS domains

Total genomic DNA was extracted according to van der Beek et al.^45^ with modifications by Park et al.^46^. After digestion using *Taq*I, *Mse*I, *Rsa*I, *Hae*III and *Alu*I restrictases, adapters for PCR^16^ were ligated to the fragments. All five digestion-ligation products per varietal DNA library were pooled and 1 µl of a pool was used for each of the 16 primer-specific PCRs. For the initial linear, target-specific amplification, the reaction contained 4 µl of 5 × HOT FIREPol Blend Master Mix (Solis Biodyne) amended with 7.5 mM MgCl_2_ and 2 µl of 0.1 µM NBS domain-specific primer (Table 1) in a total 20-µl volume. Conditions for the linear PCR followed van der Linden et al.^16^ and annealing temperature settings were specific for each primer. Subsequently, for the exponential PCR 20 µl of a master mix containing 2 µl of 10 µM NBS domain-specific primer, 2 µl of 10 µM adapter primer and 4 µl of 5 × HOT FIREPol Blend Master Mix (Solis Biodyne) with 7.5 mM MgCl_2_ and 12 µl water were added to the linear PCR product to give a total volume of 40 µl. All PCR products were cleaned using the NucleoSpin 96 PCR Clean-up kit (Macherey Nagel). The products of all 16 NBS domain-specific PCR primers per varietal library were pooled to obtain in total 96 libraries for sequencing. To estimate the fragment size distribution within each sample, we used the High Sensitivity NGS Fragment Analysis Kit (Advanced Analytical) on a Fragment Analyzer (Advanced Analytical). We had the libraries prepared and HiSeq-2000 (Illumina) sequenced by GATC Biotech AG (Germany). In brief, for library preparation, the PCR products were randomly fragmented, ligated to library-specific indices including Illumina primers and adapters and then gel purified to obtain fragments in a range of 200 to 480 bp. These 96 indexed libraries were joined into four sequencing superpools each consisting of 24 libraries, and run on the sequencer optimized for 100-bp paired-end sequencing.

### Isolation of *R2* like fragments and Illumina Miseq sequencing

Whole genome DNA of MF-II, TPS67, MT_pool_M, MT_pool_S, MT_pool_T, and the R2 late blight resistance differential (compare Table S4) was used. Profiling of NBS domains was made with the *R2* gene-specific PCR primers given in Table 1 (bottom part). Fragments were amplified as for the HiSeq sequencing described above, applying individual annealing temperatures as indicated in Table 1. The resulting PCR products were loaded onto agarose gels, run, and the range corresponding to 200 to 600 bp-fragments was cut out and used further. PCR products of each clone or library were pooled and barcoded with the Nextera XT kit (Illumina), according to the instructions. The barcoded samples were then cleaned using AMPpur beads (Agilent) according to the Illumina library preparation manual. The libraries were quality checked on an Agilent 2,100 Bioanalyzer. The amount of DNA was determined with PicoGreen dye (Thermo Fisher Science) and adjusted to 4 nM. A 10-µl volume per library was transferred to a new reaction tube and all samples were thoroughly mixed together. From this pool of libraries for MiSeq sequencing, 5 µl were mixed with 5 µl 0.2 N NaOH and incubated according to the library preparation manual (Illumina MiSeq reaction kit v. 3). Finally, after dilution to 12 pM, a 600-µl sample, together with 5% PhiX as internal control, was loaded onto the MiSeq sequencer and run for 2 × 300 bp using the standard settings.

### Annotation of NBS domain locations in the DM genome

The nucleotide sequences of the 704 most up-to-date NBS-LRR R genes on chromosomes 1 to 12 for DM v 4.03, as annotated by Jupe et al.^13^, were translated to obtain all possible open reading frames, of at least 200 amino acids (aa), using sixpack from EMBOSS v 6.6.0^47^. From the HMMER3 (v 3.1b1) software suite^48^, *hmmsearch3* was used to detect NBS domains, using the NB-ARC specific hidden Markov model (HMM) downloaded from Pfam and a mimimum score criterion of 100. The ARC domain is present in a wide array of resistance proteins including APAF-1 (apoptotic protease-activating factor-1), R proteins, and CED-4 (*Caenorhabditis elegans* death-4 protein)^49^. The detected NB-ARC aa sequences in the NBS-LRR R genes were aligned using ClustalW^50^ to build a DM R gene specific NB-ARC HMM with *hmmbuild*. The DM specific HMM was further used to re-detect NBS domains within the translated resistance genes. The coordinates of the detected NBS domains in the aa sequences were then converted to coordinates in the resistance genes. NBS domains that had genomic overlap (resulting from NBS domains detected on different open reading frames of a gene) were merged, and a final number of 576 NBS domains were obtained for this genome. Thus, using a DM R gene specific NB-ARC HMM, we confirmed 576 of Jupe et al.’s^13^ total 704 established R genes to carry an NBS domain.

### Analysis of next generation sequencing (NGS) data

#### Read mapping

The 100 bp-long paired-end reads from the HiSeq-2000 sequencing were trimmed for the Illumina TruSeq universal adaptor sequences (AGATCGGAAGAGCACACGTCTGAACTCCAGTCAC and AGATCGGAAGAGCGTCGTGTAGGGAAAGAGTGTAGATCTCGGTGGTCGCCGTATCATT) using cutadapt^51^ from either the 5′ or 3′ end of the read. Further trimming of sequences matching the primer regions was also performed to prevent introduction of false sequence variations from degeneracy in the NBS profiling primers (compare Table 1). The trimmed paired-end reads were mapped to the Potato Genome Sequencing Consortium Public Data Release of the sequenced doubled-monoploid (DM) potato clone v 4.03 genome assembly^15^ using NextGenMap^30^ v 0.4.10 at default parameters. This involves local alignment of the read (ends of reads might get clipped) with a required minimum mapped length of 0.5 fraction of the read-length and 0.65 fraction identity between the mapped read portion and DM genome to be considered mapped. The 300 bp-long paired-end reads from MiSeq sequencing were trimmed for *R2* gene-specific PCR degenerate primers sequences and mapped with NextGenMap 0.4.12 at default parameters, as described above.

#### Variant calling

Freebayes^26^ v 0.9.9 was used to detect polymorphisms within NBS domains (see next section), with respect to the DM genome with parameters of ploidy 4, min alternate allele fraction of 0.1 and minimum 10 reads. Ploidy 2 was used for the CRP (diploid) libraries.

#### Haplotype phasing

HapCompass^27^ v 0.7.7 was used for phasing of variants into haplotypes at NBS domains with parameter ploidy 12.

### Annotation and classification of novel NBS domains

We observed that a substantial number of reads were mapped to regions other than NB-LRR R genes. Therefore, we performed a search with the DM R gene-specific NB-ARC HMM in regions where we obtained at least 10 × coverage (number of reads) across a minimum length of 150 bp occurring in at least 10 cultivars. In addition, we used a tblastn search for the P-loop, GLPL, and Kinase-2 motifs (from ^12^) in these regions. With this method, we detected 11 additional, novel NBS domains that had significant results given by the HMM and tblastn search. These additional domains were located in genes not previously designated as NBS-LRR R genes by Jupe et al.^13^. To determine the completeness of the putative NBS-LRR R genes, the nucleotide sequences for the full gene were translated into all 6 possible reading frames using TranslateSequence.jar from NLR-Parser^25^ and mast from meme suite v 4.9.1^24^ was used to detect all NBS-LRR motifs published in Jupe et al.^12^. The detected motifs in the genes were then used for classification of completeness and type, using NLR-Parser^25^.

### Assigning of clusters for genes containing novel NBS domains

The genes containing the novel NBS domains were assigned cluster Ids based on criteria for clustering in^12^, which were: 1. Distance between neighboring NB-LRRs of a cluster is less than 200 kb 2. Less than eight non-NB-LRR genes are between each NB-LRR gene of the cluster. We assigned the genes containing novel NBS domains to the cluster, if present, of the next nearest NB-LRR R genes within 200 kb when there were less than 8 non-NB-LRR genes in between. As genes within a cluster are often a result of localized duplication events and have similar sequences^28^, a further check was done with a blastn search of the novel NBS sequence against all the known NBS sequences to confirm that the most similar NBS sequences (best blastn hits) to the novel NBS domain were from the same cluster. We observed that whenever we could classify a novel NBS sequence based on the above criteria by Jupe et al.^12^ among the best blast hits, the same cluster ID would also be present (see Supplementary Table S2).

### Blastn of unmapped MiSeq reads

Blastn searches of the NCBI nucleotide database (nt-db) to identify the most similar known sequences (significant blast hits with e-values smaller than 1e^-5^) to the unmapped reads were performed separately for the read categories below. Category-specific descriptions of the preprocessing of reads are included.

- Single unmapped read (other read of pair maps to the DM genome): blastn of single reads were performed without the other pair’s sequence. We found 63% of these had no blast hit while 8% of these had a blast hit to a Solanum species and 14% of these had a hit to *Triticum turgidum*.
- Paired unmapped reads (both reads do not map to the DM genome):
- The reads were first merged using PEAR v0.9.6 (paired end read merger tool)^52^. We found 14% of these had no blast hit while 9% of these had a blast hit to a Solanum species and 76% to *Triticum turgidum*.
- If pair was not merged in (a), the reads were concatenated one after another to obtain a longer sequence. The second read was reverse complemented before concatenation. We found 31% of these had no blast hit while 6% of these had a blast hit to a Solanum species and 47% to *Triticum turgidum*.
- Some of the reads were ‘discarded’ by PEAR when they had too many Ns. Reads that were made up of a long string of Ns were filtered from these discarded reads (52%), and the remaining reads were used individually for a blastn. We found 11% had no blast hit while 3% had a blast hit to a Solanum species and 13% to *Triticum turgidum*.

## Accession numbers

European Nucleotide Archive ENA, accession no. ERP086266 and study id PRJEB83917. The SolariX project can be found at www.cibiv.at/SolariX.

## Supplementary information

Supplementary Figure S1.

Supplementary Figure S2.

Supplementary Methods S1.

Supplementary Table S1.

Supplementary Table S2.

Supplementary Table S3.

Supplementary Table S4.

Supplementary Table S5.

Supplementary Table S6.

Supplementary Table S7.

## References

[1] Hawkes JG, Francisco-Ortega J (1990). The early history of potato in Europe. Euphytica.

[2] Ross H (1986). Kartoffelzüchtung.

[3] Engel FA (1987). Prehistoric Andean Ecology: Man, Settlement, and Environment in the Andes.

[4] Hawkes JG (1990). The Potato, Evolution, Biodiversity and Genetic Resources.

[5] Reader J (2009). The Untold History of the Potato.

[6] Müller KO (1951). Über die Herkunft der W-Sorten, ihre Entwicklungsgeschichte und ihre bisherige Nutzung in der praktischen Kartoffelzüchtung. Z. Pflanzenzüchtung.

[7] Foldo NE, Jellis GJ, Richardson DE (1987). Genetic Resources: Their preservation and utilization in the production of new potato varieties. The Production of New Potato Varieties: Technological Advances.

[8] Rodewald J, Trognitz B (2013). Solanum resistance genes against *Phytophthora infestans* and their corresponding avirulence genes. Mol. Plant Pathol..

[9] Black W, Mastenbroek C, Mills WR, Peterson LC (1953). A proposal for an international nomenclature of races of *Phytophthora infestans* and of genes controlling immunity in *Solanum demissum* derivatives. Euphytica.

[10] Ballvora A (2002). The *R1* gene for potato resistance to late blight (*Phytophthora infestans*) belongs to the leucine zipper/NBS/LRR class of plant resistance genes. Plant J..

[11] Trognitz BR, Trognitz FC (2007). Occurrence of the R1 allele conferring resistance to late blight in potato R-gene differentials and commercial cultivars. Plant. Pathol..

[12] Jupe F (2012). Identification and localisation of the NB-LRR gene family within the potato genome. BMC Genom..

[13] Jupe F (2013). Resistance gene enrichment sequencing (RenSeq) enables reannotation of the NB-LRR gene family from sequenced plant genomes and rapid mapping of resistance loci in segregating populations. Plant J..

[14] Lozano R, Ponce O, Ramirez M, Mostajo N, Orjeda G (2012). Genome-wide identification and mapping of NBS-encoding resistance genes in *Solanum tuberosum* Group Phureja. PLoS ONE.

[15] Xu X (2011). Genome sequence and analysis of the tuber crop potato. Nature.

[16] Van der Linden GC (2004). Efficient targeting of plant disease resistance loci using NBS profiling. Theor Appl Genet.

[17] Spooner DM, McLean K, Ramsay G, Waugh R, Bryan GJ (2005). A single domestication for potato based on multilocus amplified fragment length polymorphism genotyping. PNAS.

[18] Kuang H, Woo S-S, Meyers BC, Nevo E, Michelmore RW (2004). Multiple genetic processes result in heterogeneous rates of evolution within the major cluster disease resistance genes in lettuce. Plant Cell.

[19] Ellis J, Dodds P, Pryor T (2000). Structure, function and evolution of plant disease resistance genes. Curr. Opin. Plant Biol..

[20] Leister D (2004). Tandem and segmental gene duplication and recombination in the evolution of plant disease resistance genes. Trends Genet..

[21] Chae E (2014). Species-wide genetic incompatibility analysis identifies immune genes as hotspots of deleterious epistasis. Cell.

[22] Trognitz BR (1998). Inheritance of resistance in potato to lesion expansion and sporulation by *Phytophthora infestans*. Plant. Pathol..

[23] Nakitandwe J, Trognitz F, Trognitz B (2007). Reliable allele detection using SNP-based PCR primers containing Locked Nucleic Acid: application in genetic mapping. Plant methods.

[24] Bailey TL, Gribskov M (1998). Combining evidence using p-values: application to sequence homology searches. Bioinformatics.

[25] Steuernagel B, Jupe F, Witek K, Jones JDG, Wulff BBH (2015). NLR-parser: rapid annotation of plant NLR complements. Bioinformatics.

[26] Garrison, E. & Marth, G. Haplotype-based variant detection from short-read sequencing. arXiv:1207.3907 [q-bio] (2012).

[27] Aguiar D, Istrail S (2012). HapCompass: a fast cycle basis algorithm for accurate haplotype assembly of sequence data. J. Comput. Biol..

[28] Meyers BC, Kozik A, Griego A, Kuang H, Michelmore RW (2003). Genome-wide analysis of NBS-LRR-encoding genes in *Arabidopsis*. Plant Cell.

[29] Yang S, Zhang X, Yue J-X, Tian D, Chen J-Q (2008). Recent duplications dominate NBS-encoding gene expansion in two woody species. Mol Genet Genomics.

[30] Sedlazeck FJ, Rescheneder P, von Haeseler A (2013). NextGenMap: fast and accurate read mapping in highly polymorphic genomes. Bioinformatics.

[31] Malcolmson JF, Black W (1966). New R genes in *Solanum demissum* Lindl. and their complementary races of *Phytophthora infestans* (Mont,) de Bary. Euphytica.

[32] Trognitz, B., Trognitz, F. & Fuchs, F. Molecular markers for high-throughput selection of late blight resistant potato. In *EAPR 2011, Abstracts, 8th Triennial Conference of the. European Association for Potato Research**, *ISBN: 978-952-10-7104-1, p. 67. (2011).

[33] Feingold S, Lloyd J, Norero N, Bonierbale M, Lorenzen J (2005). Mapping and characterization of new EST-derived microsatellites for potato (*Solanum tuberosum* L.). Theoret. Appl. Genet..

[34] Milbourne D (1998). Isolation, characterisation and mapping of simple sequence repeat loci in potato. Mol Gen Genet.

[35] Michelmore RW, Meyers BC (1998). Clusters of resistance genes in plants evolve by divergent selection and a birth-and-death process. Genome Res..

[36] Andolfo G (2014). Defining the full tomato NB-LRR resistance gene repertoire using genomic and cDNA RenSeq. BMC Plant Biol..

[37] Witek K (2016). Accelerated cloning of a potato late blight–resistance gene using RenSeq and SMRT sequencing. Nat. Biotechnol..

[38] Trognitz, B. R. & Trognitz, F. Developing molecular markers for selection of resistance against late blight in potato. In *Proceedings of 60. Tagung Vereinigung Pflanzenzüchter und Saatgutkaufleute Österreichs 2009* 87–90. ISBN: 978-3-902559-37-s1. (2010).

[39] Cubillos AG, Plaisted RL (1976). Heterosis for yield in hybrids between *S*.* tuberosum* ssp. tuberosum and *Tuberosum* ssp. andigena. Am. Potato J..

[40] Huarte MA, Plaisted RL (1984). Selection for Tuberosum likeness in the vines and in the tubers in a population of Neotuberosum. Am. Potato J..

[41] Plaisted, R. L. Advances and limitations in the utilization of Neotuberosum in potato breeding. In *Production of new potato varieties: technological advances *(eds. Jellis, G. J., Richardson, D. E., 1987).

[42] Trognitz FC, Trognitz BR (2005). Survey of resistance gene analogs in *Solanum caripense*, a relative of potato and tomato, and update on R gene genealogy. Mol Gen Genom..

[43] Meyers BC (1999). Plant disease resistance genes encode members of an ancient and diverse protein family within the nucleotide-binding superfamily. Plant J..

[44] Pan Q (2000). Comparative genetics of nucleotide binding site-leucine rich repeat resistance gene homologues in the genomes of two dicotyledons: tomato and Arabidopsis. Genetics.

[45] van der Beek JG, Verkerk R, Zabel P, Lindhout P (1992). Mapping strategy for resistance genes in tomato based on RFLPs between cultivars: Cf9 (resistance to *Cladosporium fulvum*) on chromosome 1. Theoret. Appl. Genetics.

[46] Park T-H (2005). High-resolution mapping and analysis of the resistance locus Rpi-abpt against *Phytophthora infestans* in potato. Mol. Breed..

[47] Rice P, Longden I, Bleasby A (2000). EMBOSS: the European molecular biology open software suite. Trends Genet..

[48] Eddy SR (2011). Accelerated Profile HMM Searches. PLoS Comput. Biol..

[49] Van der Biezen EA, Jones JDG (1998). Plant disease-resistance proteins and the gene-for-gene concept. TIBS.

[50] Larkin MA, Clustal W, Clustal X (2007). Version 2.0. Bioinformatics.

[51] Martin M (2011). Cutadapt removes adapter sequences from high-throughput sequencing reads. EMBnet J..

[52] Zhang J, Kobert K, Flouri T, Stamatakis A (2014). PEAR: a fast and accurate Illumina paired-end reAd mergeR. Bioinformatics.

[53] Pel MA (2009). Mapping and cloning of late blight resistance genes from *Solanum venturii* using an interspecific candidate gene approach. Mol. Plant Microbe Interact..

