# Supplementary material for: A compendium of genome-wide sequence reads from NBS (nucleotide binding site) domains of resistance genes in the common potato
Source: Sci Rep. 2020 Jul 9;10:11392. doi: 10.1038/s41598-020-67848-z (PMC7347568; doi:10.1038/s41598-020-67848-z)

Supplementary Figure S1.

Blastn search to the NCBI nucleotide database for 648 960 unmapped reads (38% of all reads) of the MiSeq library for MFII. 62.88 % of these unmapped reads had a significant blast hit (e value smaller than 1e-5): 7.72 % to the Solanum genus and 44.24% in the species *Triticum turgidum.* Most of the hits were to nucleotide sequences representing R genes: 4.52% in the Solanum genus and all 44.24% in *T. turgidum.*

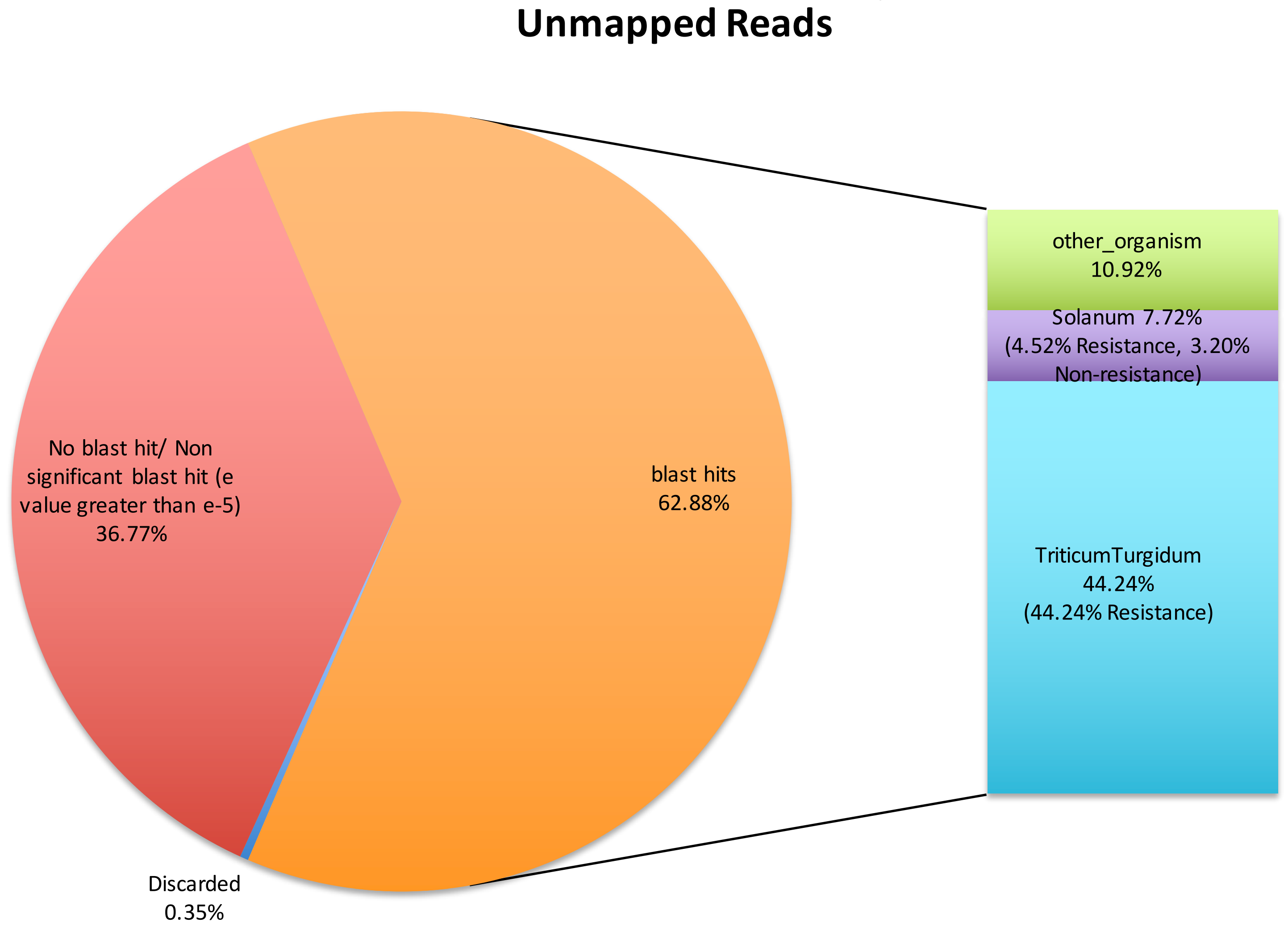

Supplement: Supplementary file 1 — Supplementary Figure S1. [file 41598_2020_67848_MOESM1_ESM.pdf]
