# Supplementary material for: A compendium of genome-wide sequence reads from NBS (nucleotide binding site) domains of resistance genes in the common potato
Source: Sci Rep. 2020 Jul 9;10:11392. doi: 10.1038/s41598-020-67848-z (PMC7347568; doi:10.1038/s41598-020-67848-z)

Supplementary Figure S2.

Inferring genetic similarity between cultivars based on variations in read coverage for nucleotide binding site domains. Read coverage frequencies (RCF), by resistance (R) locus on the reference genome DM, of all 96 cultivars represented as a heat map; the uncompressed version of Figure 6.

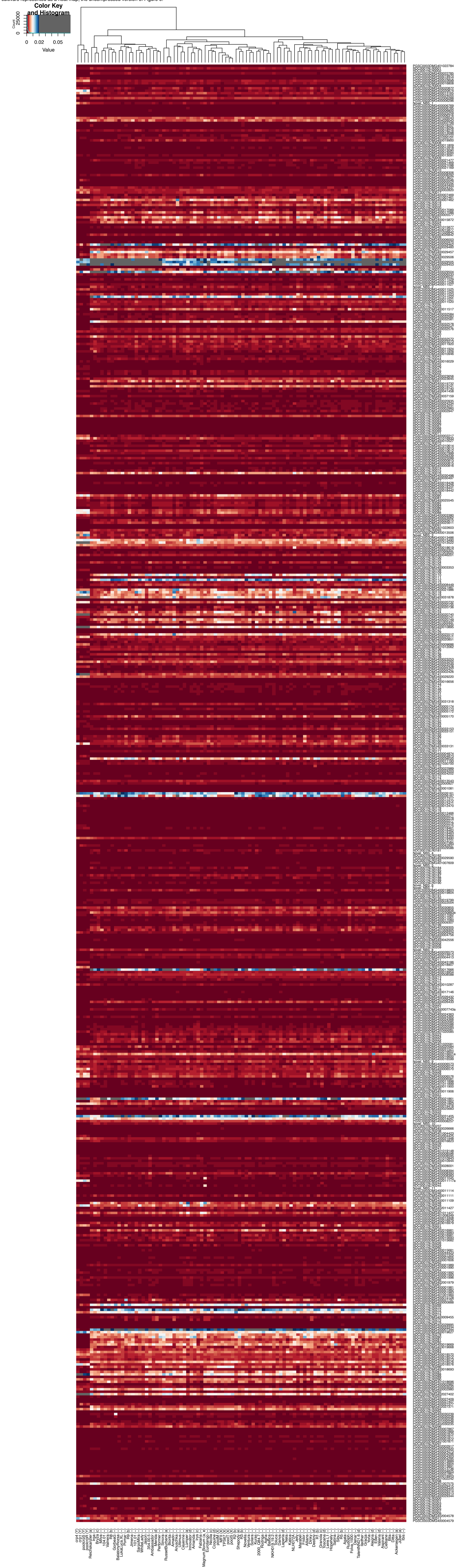

Supplement: Supplementary file 2 — Supplementary Figure S2. [file 41598_2020_67848_MOESM2_ESM.pdf]
