# Supplementary material for: A compendium of genome-wide sequence reads from NBS (nucleotide binding site) domains of resistance genes in the common potato
Source: Sci Rep. 2020 Jul 9;10:11392. doi: 10.1038/s41598-020-67848-z (PMC7347568; doi:10.1038/s41598-020-67848-z)
Supplement: Supplementary file 3 — Supplementary Methods S1. [file 41598_2020_67848_MOESM3_ESM.docx]

**Supplementary Methods S1.** Development of CAPS (Cleaved Amplified Polymorphic Sequence) markers for the distinction of disease resistant and susceptible progenies of a population of CRP segregating for resistance to late blight.

SNPs observed on the Illumina reads for locus PGSC0003DMG402020585 were chosen to design specific primers based on the DM genome reference that could amplify exclusively this locus. Two primers, 2020585_F (TGTTCCGTTCTTTCTCTTGC) and 2020585_R (CATTCCATTGGACCAGAGAG), were applied in a 50-µl PCR reaction containing 10 µl of Blend Master Mix (Solis BioDyne), 0.75 µM of each primer and 100 ng of genomic DNA. The PCR was set to an initial 15-min step at 95°C, followed by 35 cycles of 95°C for 20 sec, 57°C for 30 sec and 72°C for 1 min. PCR products were visualized on standard 1% agarose gels. For verification of the predicted SNP-dependent restriction sites (allelic differences) the PCR product was cut with *Dde*I (New England Biolabs) or *Tai*I (Thermo Fisher Scientific) in a 15-µl reaction containing 10 µl of the PCR product, 1x the corresponding digestion buffer and 5 U of the restriction enzyme. The mixture was incubated for two hours at 37°C for *Dde*I and at 65°C for *Tai*I. Digestion products were run and scored on standard 2% agarose gels.

**Supplementary Methods S2.** Generation of a draft AB (Alegria x Baltica) linkage map and fine-mapping of Alegria chromosome IX.

For framework genetic mapping of the PVY resistance, a set of 45 potato-specific Simple Sequence Repeat (SSR) markers (Supplementary Table S4), three or four per chromosome, was applied on samples of genomic DNA of all 250 AB progenies and the parents, kindly provided by NORIKA (Nordring-Kartoffelzucht-und Vermehrungs-GmbH, Groß Lüsewitz, Germany) following the procedure of Milbourne *et al.*^34^. In brief, PCR was performed in a 20-µl reaction volume containing 1 U FIREPol polymerase (Solis Biodyne), 1x BD buffer, 0.2 mM dNTPs (Solis Biodyne) and 0.5 µM of each (forward and reverse) primer. The cycling conditions were 1 min at 95°C followed by 30 cycles of 1 min at 94°C and 1 min at the corresponding primer-specific annealing temperature, and finally 1.5 min at 72°C. The forward primer was 5’-end fluorescence labeled (FAM or HEX, Sigma), and 1 µl of the 1:25 or 1:50 diluted amplicon was mixed with 0.3 µl 350 ROX size standard and 10 µl HiDi formamide (Applied Biosystems) and run on an AB3100 automated sequencer (Applied Biosystems) equipped with a 22-cm capillary, Pop4 polymer and settings; IV 6, IT 3, 15 V, run time 600. The resulting data was analyzed in Genotyper_3.7_0125 software (Applied Biosystems). Genetic linkage maps were calculated based on the segregating markers detected, using the Tetraploid Map program package^54^ with default settings. The SSR markers yielded haplotypes segregating 1:0 (22 markers), 3:1 (20), 5:1 (2) and 35:1 (2) and these, together with the qualitative phenotype of response to PVY (provided by NORIKA) scored as a single dominant marker, were used to generate a genetic framework map of cv. Alegria.

**Fine mapping of cultivar Alegria chromosome IX**

PCR primers were designed based on single nucleotide polymorphisms (SNPs) distinguishing the NBS tags for chromosome IX of cvs. Alegria and Baltica, as obtained via HiSeq-2000 sequencing, relative to the reference genome; DM v 4.03. All primers were made to detect just Alegria-specific SNPs. A SNP to be analyzed should occur in cv. Alegria and not in Baltica and optimally it should occur as an allele in simplex frequency. Therefore, the total of fragments carrying an alternate vs. the reference base at the SNP position should fit a 1:3 ratio (as determined by a chi-square test for goodness-of-fit), relative to the total number of reads analyzed for that genomic position of cv. Alegria (minimum 20 reads covering the SNP). The mapped position of the SNPs was visualized in the IGV Interactive Genome Viewer^55^.

Allele-specific primers containing the selective SNP at their outer 3’ position were designed using the Primer3 online tool^56^. Mismatches were introduced at the second position adjacent to the selective nucleotide at the 3’ end to boost primer selectivity following the criteria described by Liu et al.^57^. Allele-specific PCRs were performed on a C1000 thermocycler (BioRad). Optimal reaction conditions for each primer pair were determined via gradient PCR using 0.5 µM of each primer, primer-specific concentrations of MgCl_2_ and dNTP as given in Supplementary Table S6, 1.25 U HOT FIREPol polymerase (Solis BioDyne) and 10 ng of DNA in a total 20-µl volume. Cycling conditions were; 15 min at 95°C, followed by 40 cycles of 30 sec at 95°C, 30 sec at the corresponding annealing temperature of the individual primer, and 1 min at 72°C, and a final elongation during 10 min at 72°C. Amplification products were assessed on standard 1% agarose gels.

34. Milbourne, D. *et al.* Isolation, characterisation and mapping of simple sequence repeat loci in potato. *Mol Gen Genet* **259**, 233–245 (1998).

54. Hackett, C. A. & Luo, Z. W. TetraploidMap: Construction of a linkage map in autotetraploid species. *Journal of Heredity* **94**, 358–359 (2003).

55. Thorvaldsdóttir, H., Robinson, J. T. & Mesirov, J. P. Integrative Genomics Viewer (IGV): high-performance genomics data visualization and exploration. *Briefings in Bioinformatics* **14**, 178–192 (2013).

56. Koressaar, T. & Remm, M. Enhancements and modifications of primer design program Primer3. *Bioinformatics* **23**, 1289–1291 (2007).

57. Liu, J. *et al.* An improved allele-specific PCR primer design method for SNP marker analysis and its application. *Plant Methods* **8**, 1–9 (2012).
